# Investigating the economic case of a service to support carers of people with dementia: A cross‐sectional survey‐based feasibility study in England

**DOI:** 10.1111/hsc.12799

**Published:** 2019-06-21

**Authors:** Francesco Longo, Rita Faria, Gillian Parker, Kate Gridley, Fiona Aspinal, Bernard Van den Berg, Helen Weatherly

**Affiliations:** ^1^ Centre for Health Economics University of York York UK; ^2^ Social Policy Research Unit University of York York UK; ^3^ NIHR CLAHRC North Thames, Department of Applied Health Research University College London London UK; ^4^ Department of Health Sciences VU University Amsterdam The Netherlands

**Keywords:** Admiral Nursing, carers, costs, economic evaluation, outcomes, social care

## Abstract

Carers contribute essential support to enable people with dementia to continue living within the community. Admiral Nurses provide specialist dementia support for carers of people with dementia, including offering expert emotional support and guidance, and work to join up different parts of the health and social care system to address needs in a co‐ordinated way. The cost‐effectiveness of this service is not clear. We undertook a feasibility study to explore related outcomes and costs for these carers. A cross‐sectional, clustered survey was undertaken in England in 2017, in areas with and without Admiral Nursing (AN). The survey questionnaire included questions on the characteristics of the carers and the person with dementia, outcomes (care‐related quality of life [CRQoL], self‐efficacy and subjective well‐being), use of health and social care services, out‐of‐pocket costs and time spent on informal care. We used different econometric techniques to compare the outcomes and the costs of the carers with and without AN services: linear regression, propensity score matching and instrumental variables analysis. These techniques allowed us to control for differences in observed and unobserved characteristics between the two groups of carers which determined outcomes and costs. We concluded that AN services might have a positive effect on carers' CRQoL, self‐efficacy and subjective well‐being. Furthermore, we found little difference in costs between carers using AN and those using usual care, or in the costs of the people with dementia they care for. Our findings provided an initial indication as to whether AN services could be good value for money. The key limitation of the study was the difficulty in controlling for unobserved characteristics because of the cross‐sectional nature of our observational data. To diminish this limitation, our survey could be used in future studies following carers with and without AN services over time.

**What is known about the topic**

- The Admiral Nursing (AN) service provides carers with expert emotional support and guidance.
- One of the first evaluations of AN found that carers using AN had similar general health and survival compared to carers not using AN.
- A 2013 systematic review concluded that quantitative evaluations of AN on outcomes and costs are sparse.

**What this paper adds**

- This is the first time that outcomes and costs have been compared between carers with and without AN.
- The outcomes of carers using AN were similar if not better than their counterparts without access to AN services.
- The costs of health and social care services were similar between the two groups.

## 1 INTRODUCTION

Carers contribute essential support to enable people with dementia to continue living within the community. Admiral Nursing (AN), supported by the charity Dementia UK, is the only specialist nursing service with a specific focus on supporting carers of people with dementia. This service provides carers with expert emotional support and guidance, and aims to join up different parts of the health and social care system to address needs in a co‐ordinated way.

Provision of AN services is diverse across England and Wales, and sometimes depends on financial support from charitable grants. If AN services are to be commissioned across the country and paid for by the public sector, information is needed on their outcomes and costs. To date, there is little quantitative evidence on the outcomes and costs of the AN service. One of the first evaluations of the service compared the mental health of carers using the service (*n* = 43) with those without the AN service (*n* = 61), based on the general health questionnaire (Woods, Wills, Higginson, Hobbins, & Whitby, 2003). It found that carers with AN support had better outcomes on anxiety and insomnia but similar levels of general health and survival. A 2013 systematic review concluded that the literature is sparse in terms of evaluating the outcomes and costs associated with AN service, but that carers were satisfied with the AN service and they valued its support (Bunn, Pinkney, Drennan, & Goodman, 2013).

To address this evidence gap, we investigated the outcomes and costs of AN services on carers compared to usual care for carers not using the AN service. The primary objective of the main study, of which this economic study is a part, was to test the acceptability and feasibility of surveying carers of people with dementia through a self‐administered questionnaire including questions on their characteristics, outcomes and costs. The findings of our study could inform the design and implementation of a full‐scale evaluative study and could provide early‐stage information to commissioners on whether AN services might be effective and cost‐effective. The findings reported here contribute to the expanding evidence base, to inform practitioners and policy makers about assessment of the effectiveness and costs of services that support carers of people with dementia.

## 2 METHODS

### 2.1 Selection of local authorities and recruitment of carers

To recruit AN carers, we selected 16 AN services that were not involved in a concurrent evaluation, had a minimum caseload of 35 carers as of September 2016, were serving people living in the community and where any dementia carer could use the service. To recruit non‐AN carers, we chose local authorities that did not provide AN using the Adult Social Care Efficiency Tool (ASCET; Department of Health & Social Care, 2015). The ASCET allowed us to match local authorities providing AN with local authorities without AN on social care expenditure and outcomes, where outcomes were captured through the Adult Social Care Outcome Framework indicators, such as social care‐related quality of life (CRQoL) and the proportion of people having control over daily life, for older people and people with learning disabilities. The ASCET allows meaningful comparisons between local authorities on social care expenditure and outcomes by controlling for factors outside the control of local authorities including: proportion of people aged 65 years and older; life expectancy at 65 years for women; proportion of people over 65 receiving income support, pension credit, or job seekers allowance; proportion of homeless people; population density; proportion of households in social rented accommodation; proportion of males over 65, and area cost adjustment.

We took a pragmatic approach to sample size calculation using the effect sizes from a randomised controlled trial of community occupational therapy in the Netherlands (Graff et al., 2007), given that we did not have commensurate data relating to AN. To run a multivariate analysis controlling for approximately 20 variables, we calculated that a sample of 320 participants would be enough to detect differences of the size observed by Graff et al. (2007).

In local authorities with AN, the survey was sent out to those carers for whom the AN providers held postal or email addresses. In local authorities without AN, national and local ‘voluntary sector' groups for carers and people with dementia were contacted to disseminate the survey. Carers residing in local authorities with the AN service and without it (for simplicity, respectively, AN and non‐AN carers from now on) received the request to complete the survey at one point in time between January and March 2017.

### 2.2 Development of the self‐administered survey

The survey was developed for self‐completion by the carers through an online or postal questionnaire. The questionnaires collected information on the characteristics of the carer and the person with dementia, carer quality of life, use of health services by the carer and the person with dementia, and use of social care services by the person with dementia. Additionally, we asked about the carer's use of any carer‐specific services such as carers' groups or advice services.

Selection of the outcome measures was informed by focus groups and interviews with carers. Through these, the outcomes that the carers thought were most influenced by the quality and level of support they received or might receive from specialist support services were CRQoL, self‐efficacy, and mental and physical health. Additionally, we included measures of well‐being because they were likely to be relevant to the policymaker. After cognitive interviewing and piloting with carers, the questionnaire was revised. The survey questionnaire is available in the full report of the research.

Prior to sending out the questionnaire, we tested it through cognitive interviews with nine carers to explore understanding and acceptability of the questions. In addition, we assessed how best to administer the questionnaire, and whether the questionnaire was sufficiently comprehensible, having sought advice from our virtual advisory group members and steering group members.

### 2.3 Instruments to measure carer outcomes

Having identified the outcomes above, we explored the international literature and, chose, guided by the work of INTERDEM (Moniz‐Cook et al., 2008), what appeared to be the best measure of each outcome.

For CRQoL we chose ASCOT‐Carer. This is a validated instrument for measuring CRQoL of informal unpaid carers who care for adults with a variety of long‐term conditions, disability or problems related to old age (Malley, Fox, & Netten, 2010; Rand, Malley, Netten, & Forder, 2015; Smith, Fox, & Holder, 2009). ASCOT‐Carer covers seven domains: spending time on valued or enjoyable activities, having control over daily life, looking after oneself, feeling safe, having social contact, having space and time to be oneself and feeling encouraged and supported in the caring role (Rand et al., 2015). Each domain has four response categories from ‘no needs' to ‘high levels of needs'. An Adult Social Care Outcomes Toolkit (ASCOT)‐Carer score involves summing the answers to the seven domains, giving a range from nought (lowest CRQoL) to 21 (highest CRQoL).

We measured self‐efficacy using the caregiver self‐efficacy for managing dementia (SEMD) tool (Fortinsky, Kercher, & Burant, 2002). It includes two domains comprising the carers' confidence in managing the dementia symptoms and their confidence and experiences in using support services. The former comprises five questions with answers on a 10‐point scale, where one represents ‘not at all certain' and 10 represents ‘very certain'. For this domain, a summed score can be derived by summing the question scores with a possible range from five (least self‐efficacy) to 50 (greatest self‐efficacy). The domain on support services use is based on four questions with answers on the same 10‐point scale. The summed score for this domain has a range from four (least self‐efficacy) to 40 (greatest self‐efficacy).

We captured mental and physical health through EQ‐5D‐5L. EQ‐5D‐5L is recommended by the National Institute for Health and Care Excellence (NICE) for use in economic evaluations of health and social care interventions in the UK (NICE, 2014). It comprises five dimensions: mobility, self‐care, usual activities, pain/discomfort and anxiety/depression (Rabin & de Charro, 2001). Each dimension is described on five levels: no problems, slight problems, moderate problems, severe problems and unable to/extreme problems. EQ‐5D‐5L thus describes 3,125 possible health states, which can be converted into a preference‐based score anchored at nought for death to one for full health using a national tariff (Devlin, Shah, Feng, Mulhern, & van Hout, 2018). The preference‐based score reflects the preference for one health state over another. It ranges from −0.281 (for extreme problems on all dimensions) to 1 (no problems on any dimensions). Although the improvement in health‐related quality of life (HRQoL) appeared to be one of the carers' expected outcomes, AN support aims to help carers to ‘cope' rather than to increase their HRQoL per se. We therefore excluded HRQoL from the set of outcomes of interest and instead used it to capture carer health as an additional measure of needs within the econometric analysis.

To measure subjective well‐being, carers were asked how satisfied they were with their life nowadays, and about their happiness yesterday. Both approaches use a scale of nought to 10, with nought meaning not at all satisfied (or unhappy) and 10 meaning completely satisfied (or completely happy). These questions are used in the Office of National Statistics Annual Population Survey (ONS, 2011) and have also been used in previous studies of informal carers (Van den Berg & Ferrer‐i‐Carbonell, 2007; Van den erg, Fiebig, & Hall, 2014).

### 2.4 Resource use and costs

We took a broad perspective to investigate the economic case for AN. We costed resource use falling on the NHS, social care services, voluntary services, out‐of‐pocket costs and time spent caring (informal unpaid care). We measured service use by carers and the person with dementia (reported by the carer) such as specialist support services for carers (including AN), healthcare, social care and voluntary sector services, as well as any out‐of‐pocket costs incurred in accessing or using associated services. In order to reduce recall bias, questions on resource use referred to the past 4 weeks.

We costed resource use using nationally available unit costs (Curtis & Burns, 2016; Department of Health, 2016; Glendinning et al., 2010) to aid transferability of results. Costs relate to the financial year 2015/16. Unit costs are presented in Tables S1 and S2. The cost dependent variable of key interest is a measure of overall costs calculated as the sum of health and social care costs for both carers and care recipients, including the cost of AN. This assumes that the cost of AN falls on the health and social care budget, although this may not always be the case and may vary across local authorities. Health and social care costs are calculated by multiplying the amount of resources used in the past 4 weeks by the relevant unit cost (Table S1), and they do not include any out‐of‐pocket or informal care costs.

In addition, we quantify out‐of‐pocket costs and informal care costs. The out‐of‐pocket costs were self‐reported by the respondent and they referred to the use of services other than the AN service, such as voluntary sector or social care services. Time spent in caring for the person with dementia was costed using the proxy good method (Van den Berg et al., 2006; Van Berg & Spauwen, 2006; Weatherly, Faria, & Van Den Berg, 2014; Weatherly et al., 2017). This method values informal care time with the market price of a close substitute for a specific care task (Table S2). To obtain information on care time, respondents were asked to indicate which care tasks they carried out from a list of 10 different tasks (obtained from the 2009 ‘survey of carers in households'; NHS Information Centre, 2010), and the amount of time spent on each of the indicated tasks. Where people indicated that they were involved in three or more tasks, we asked them to provide the information about hours of care for the three tasks that had taken up the most time. Finally, we asked carers to record how much time they had spent caring overall, in the previous 24 hr.

### 2.5 Observed confounders

Our qualitative work suggested that AN services tend to target carers with greater needs for support. Carers with greater needs were more likely to self‐refer themselves to the AN service. Given the observational nature of our study, AN carers were likely to have different characteristics compared to non‐AN carers. A direct or unadjusted comparison between AN and non‐AN carers may highlight differences in outcomes and costs driven by the different carers' characteristics, rather than the use of AN. To account for potential sources of confounding, the survey included questions on the characteristics of the carer and the characteristics of the person with dementia. Carer characteristics comprised: gender, age, education, work situation, household financial difficulties, whether the carer was a sole carer, relationship with the care recipient, type and amount of time of care provided, number of years caring, and availability of a replacement for a break. HRQoL measured using EQ‐5D‐5L was used as a control variable in the analysis, rather than a dependent variable. Additionally, we collected information on care recipient characteristics including age, duration of symptoms of dementia, existence of a formal diagnosis, type of dementia such as Alzheimer, vascular dementia, or other type of dementia, and perceived severity of dementia (categorised from moderate to high severity). As these characteristics were observed, they are called observed confounders.

### 2.6 Econometric analysis of outcomes and costs

The dependent variables in our econometric analyses are outcomes including ASCOT‐Carer score, SEMD score on both management of dementia symptoms and service use domains, overall life satisfaction and happiness yesterday, and costs including overall costs (see Section 2.4), carer and care recipient healthcare costs, and social care costs. We controlled for observed characteristics in the carers and care recipients (the observed confounders) using linear regression analysis and propensity score matching (PSM).

Linear regression analysis provides unbiased estimates of the effect of the AN service if the regression includes all the characteristics which affect outcomes, costs, and the decision to use AN services, where the relationship between these characteristics and the outcomes or costs is constant (Wooldridge, 2015).

Propensity score matching matches AN and non‐AN carers given their propensity score (Rosenbaum & Rubin, 1985). The propensity score is the conditional probability of receiving the AN service given the observed confounders. It was obtained by regressing whether or not carers were AN carers on the observed confounders (see Section 2.5) using a logit regression. We then matched AN and non‐AN carers using the kernel technique, which compares each AN carer with a counterfactual constructed as the kernel weighted average of multiple individuals in the control group. We used the Epanechnikov kernel function although the choice of the kernel function tends, in practice, not to make a difference (Caliendo & Kopeinig, 2008; DiNardo & Tobias, 2001). The counterfactual mostly depends on the distance between propensity scores of the treated and untreated individual within a specific bandwidth (Pagan & Ullah, 1999; Silverman, 2018). Following Heckman, Ichimura, and Todd (1997) and Garrido et al. (2014), we set the bandwidth to 0.06 to optimise the trade‐off between variance and bias of the kernel estimator. PSM provides unbiased estimates of the effect of the AN service if, after matching, AN and non‐AN carers have a similar probability of receiving the AN service (i.e. a similar propensity score) and there are no unobserved confounders. The difference in outcomes and costs between the matched groups represents the effect of the AN service in the carers who received AN. We assessed the validity of the PSM by checking the balance of the covariates, standardised differences and visual inspection.

Instrumental variable (IV) regression accounts for any unobserved characteristics that determine outcomes, costs, and the use of AN services, such as resilience and ability to care. This econometric approach can deal with these unobserved characteristics through a variable, the instrument, that is correlated with having AN services but has no direct effect on outcomes and costs, and is not correlated with unobserved characteristics that affect costs and outcomes. The difference in outcomes and costs obtained with the IV analysis represents the effect of the AN service in those carers who were induced to take up the AN service due to the instrument.

We explored two IVs: travel time by car between the carer and the nearest AN service and type of local authority classified as County, London, Metropolitan and Unitary local authority. Travel time is likely to be correlated with accessing AN services (the further away the service the less likely its use) but it is unlikely to directly affect outcomes and health and social costs. We selected type of local authority following Forder, Malley, Towers, and Netten (2014), who argued that it determines the local authority's culture and, in turn, the local authority's propensity to invest in services for carers. Some local authorities will therefore be more willing to fund AN than others, but the culture will not have a direct effect on carer's outcomes. We tested the strength of each instrument with the Cragg‐Donald *F* statistic (Cragg & Donald, 1993).

To deal with missing data we used complete case analysis, thus removing observations with missing data. This assumes that data were missing not at random. In both the regression analysis and the IV approach we estimated robust standard errors. Since carers from the same local authority may exhibit correlations between each other, as a sensitivity analysis, we clustered standard errors within local authorities. In addition, we re‐estimated linear regressions by including local authority random effects. As a further sensitivity analysis, we estimated alternative econometric models including generalised linear model (GLM) with log link and normal or gamma distribution for outcomes and two‐part model for costs. Regressions including local authority random effects and GLM models used likelihood‐based estimators, which assumed that data were missing at random conditional on the variables included. Finally, to investigate whether our models were over fitted, we estimated linear regressions, PSM, and IV regressions using a more parsimonious specification. We carried out all analyses in Stata 15.

## 3 RESULTS

### 3.1 Response and characteristics of study sample

Calculating an overall response rate for our survey is impossible. While we know how many paper questionnaires we sent to control area third sector organisations, we do not know how many they distributed. Furthermore, while we know which organisations we sent the electronic survey, we do not know how many people received the link, nor how many people chose to open it. Also, we do not know how many carers of people with dementia potentially lived in the local authorities with and without AN services as these data do not currently exist. Twenty‐six per cent of the paper questionnaires we distributed to the AN services and third sector organisations were returned to us and were in scope. For the two organisations where we knew how many links were sent to carers, 25% and 43% of carers provided in‐scope responses. In total, we received 346 completed questionnaires which were in scope—158 (46%) were from AN service users in our selected areas and 188 (54%) were from carers in non‐AN areas.

### 3.2 Outcomes of AN and non‐AN carers

Table 1 reports the descriptive statistics for carer outcomes before controlling for the observed confounders. CRQoL using ASCOT‐Carer was 10.1, on average, and was statistically significantly lower (worse) for AN carers versus non‐AN carers (9.6 vs. 10.6) at the 5% level. This is a small difference (i.e. 1% of the average). AN carers reported significantly lower life satisfaction (4.3 vs. 5) which is a small difference on a scale from zero to 10. Self‐efficacy on symptoms management was on average 27.4 and self‐efficacy on service use 22.3. AN and non‐AN carers were statistically similar on both measures of self‐efficacy. AN carers were typically as happy as non‐AN carers.

**Table 1:** Descriptive statistics on outcomes

Outcome	All carers					AN		Non‐AN		AN versus non‐AN			
	Obs	Mean	*SD*	Min	Max	Obs	Mean	Obs	Mean	Diff	*p*‐value	95% CI	
ASCOT‐Carer score	317	10.1	4.0	0	21	147	9.6	170	10.6	−1.0	0.019**	0.166	1.870
Self‐efficacy on symptoms management	310	27.4	10.5	5	50	142	26.6	168	28.0	−1.4	0.238	−0.943	3.791
Self‐efficacy on service use	302	22.3	9.3	4	40	137	22.5	165	22.0	0.5	0.654	−2.676	1.681
Overall life satisfaction	330	4.7	2.3	0	10	153	4.3	177	5.0	−0.7	0.008***	0.187	1.216
Happiness yesterday	328	5.0	2.5	0	10	154	4.8	174	5.1	−0.3	0.278	−0.241	0.841
EQ‐5D‐5L score	330	0.775	0.181	0	1	153	0.744	177	0.802	−0.058	0.004***	0.018	0.098

Note

Standard errors of the unadjusted mean difference were bootstrapped with 1,000 replications.

Abbreviations: AN, Admiral Nursing; CI, confidence intervals; Diff, unadjusted mean difference between AN and non‐AN carers; Mean, unadjusted mean; Obs, number of observations; *SD*, standard deviation.

* *p*‐value < 0.1,

** *p*‐value < 0.05,

*** *p*‐value < 0.01.

### 3.3 Costs of AN and non‐AN carers

Table 2 reports descriptive statistics on the costs before controlling for the observed confounders. Over 4 weeks, the average cost of health and social care services of the carer‐care recipient dyad was approximately £1,000. This includes £36 for the AN service, £239 for healthcare services for the carer, £324 for healthcare services for the care recipient, and £627 for social care services. The costs varied widely. There were some differences in the cost of AN carer‐care recipient dyads versus non‐AN, although these differences were not statistically significant.

**Table 2:** Descriptive statistics on costs

Costs	All carers					AN		Non‐AN		AN versus non‐AN			
	Obs	Mean	*SD*	Min	Max	Obs	Mean	Obs	Mean	Diff	*p*‐value	95% CI	
Overall costs	260	999	1,327	0	7,000	121	1,047	139	958	89	0.567	−216	394
Cost of AN for carers	323	36	72	0	440	135	86	188	0	86	<0.001*	71	101
Carer's healthcare costs	306	239	841	0	9,110	150	198	156	277	−79	0.393	−260	102
Carer's hospital costs	317	309	1,506	0	17,932	153	221	164	391	−170	0.304	−494	154
Carer's community costs	310	28	37	0	238	151	30	159	26	4	0.338	−4	12
Care recipient's healthcare costs	297	324	837	0	6,940	148	290	149	358	−69	0.483	−261	123
Care recipient's hospital costs	308	383	1,071	0	9,206	151	372	157	393	−21	0.857	−249	207
Care recipient's community costs	305	40	47	0	273	150	42	155	37	4	0.419	−6	15
Social care costs	307	627	1,096	0	6,928	144	663	163	594	69	0.588	−180	317
Other costs													
Out‐of‐pocket costs													
Short respite/break	14	240	305	8	850	10	297	4	97	200	0.101	−39	438
Day sitting	27	37	21	6	100	12	31	15	41	−9	0.222	−25	6
Support group	5	6	2	3	8	2	7	3	6	0	0.914	−3	3
Day care centre	65	40	27	5	130	35	34	30	47	−14	0.029**	−26	−1
Other day care service	19	15	15	3	55	6	13	13	16	−3	0.585	−14	8
Home care	55	29	36	1	213	20	13	35	38	−24	0.002***	−36	−8
Meals	23	10	9	3	40	14	11	9	8	3	0.320	−3	10
Memory café	24	7	8	2	40	1	6	23	7	−1	0.712	−4	3
Informal care costs	323	459	427	25	3,181	149	437	174	477	−40	0.393	−130	51

Note

Standard errors of the unadjusted mean difference were bootstrapped with 1,000 replications.

Abbreviations: AN, Admiral Nursing; CI, confidence intervals; Diff, unadjusted mean difference between AN and non‐AN carers; Mean, unadjusted mean; Obs, number of observations; *SD*, standard deviation.

* *p*‐value < 0.1,

** *p*‐value < 0.05,

*** *p*‐value < 0.01.

The largest out‐of‐pocket cost was for short respite or break services (£240), followed by day care centre for the person with dementia (£40) and day sitting (£37). Other costs include home care (£29), other day care service (£15), meals (£10), memory café (£7) and support group (£6). The out‐of‐pocket costs for services are similar between AN and non‐AN carers apart from the cost of day care centre (£34 for AN carer vs. £47 for non‐AN carer, *p* = 0.029) and the cost of home care (£13 vs. £38, *p* = 0.002).

On average, carers had spent 12 hr providing informal care over the previous 24 hr. Using the top three informal care tasks carried out in the previous 24 hr to cost the care at its closest market replacement value, the cost was £459 on average. There was no statistically significant difference in these costs between AN and non‐AN carers.

### 3.4 Regression analysis

We used linear regressions to estimate the effect of AN on outcomes and costs after controlling for the observed confounders (Table S3; Figure S1). Table 3 reports the adjusted difference in outcomes and costs between AN and non‐AN carers based on linear regression, PSM and IV regression. The linear regression results suggest that being an AN carer was always associated with better outcomes, although the differences were not statistically significant (except for the self‐efficacy measure on service use which is weakly significant at the 10% level). This suggests that AN carers had similar levels of CRQoL, self‐efficacy and happiness as did non‐AN carers. There was no statistically significant association between being an AN carer or not, and costs. Full results of the regression analysis are reported in Table S4. Similar results were obtained when standard errors were clustered within local authorities or local authority random effects were added to the model (Table S5). This was due to a generally low intraclass correlation varying between 0.029 (for overall satisfaction) and 0.186 (for the care recipient's health care costs). Results were robust to alternative econometric models, including GLM with log link and normal or gamma distribution for outcomes, and two‐part model for costs (Table S6). Similar estimates obtained through the model including local authority random effects and GLM suggested that our results were robust to the assumption that data were missing at random (Table S7). Finally, results were robust also to a specification including fewer control variables (Table S8).

**Table 3:** Analysis of outcomes and costs

	ASCOT‐Carer score		Self‐efficacy on symptoms management		Self‐efficacy on service use		Overall life satisfaction		Happiness yesterday		Overall costs		Carer's healthcare costs		Care recipient's healthcare costs		Social care costs	
Regression																		
Coeff	0.382		1.243		1.990*		0.087		0.433		27		−170		−97		58	
Std Err	(0.397)		(1.317)		(1.060)		(0.266)		(0.279)		(203)		(145)		(133)		(146)	
95% CI	−0.400	1.163	−1.351	3.837	−0.098	4.078	−0.437	0.612	−0.117	0.983	−374	427	−457	116	−359	165	−230	346
Propensity score matching																		
Coeff	0.648		1.618		2.634**		0.171		0.575*		−113		−207		−186		11	
Std Err	(0.562)		(1.505)		(1.328)		(0.333)		(0.346)		(216)		(145)		(143)		(144)	
95% CI	−0.453	1.749	−1.332	4.568	0.031	5.238	−0.481	0.823	−0.103	1.254	−536	310	−492	77	−466	94	−272	293
Instrumental variables																		
Coeff	1.462*		2.871		3.276		0.249		0.989		−85		−27		183		−486	
Std Err	(0.854)		(3.130)		(2.633)		(0.658)		(0.636)		(424)		(220)		(235)		(326)	
95% CI	−0.212	3.136	−3.264	9.005	−1.885	8.438	−1.042	1.539	−0.258	2.236	−917	747	−459	405	−278	645	−1125	153

Note

In the regression analysis, we controlled for carer characteristics (including gender, age, education, work situation, household financial difficulties, whether the carer was a sole carer, relationship with the care recipient, type and amount of time of care provided, number of years caring, availability of a replacement for a break, and HRQoL) and care recipient characteristics (including age, duration of symptoms of dementia, existence of a formal diagnosis, type of dementia such as Alzheimer, vascular dementia, or other type of dementia, and perceived severity of dementia). We used the same independent variables in the logit regression for the calculation of the propensity score to be used in the Propensity Score Matching. Similarly, we controlled for the same independent variables in the IV regression, for which the instrument was the travel time to the closest AN provider.

No adjustment for multiple testing was implemented because of the feasibility nature of this study.

Abbreviations: AN, Admiral Nursing; CI, confidence intervals; Coeff, estimated coefficient on the Admiral Nursing dummy; HRQoL, health‐related quality of life; IV, instrumental variable; Std Err, robust standard errors.

* *p*‐value < 0.1,

** *p*‐value < 0.05,

*** *p*‐value < 0.01.

### 3.5 Propensity score matching

We constructed the propensity score by regressing whether or not carers were AN carers on the observed confounders using a logit regression. Carers taking care of a person with vascular dementia had twice the odds of being in the AN group compared to carers of people with Alzheimer's disease. Carers with Master's or higher degrees had 15%–23% lower odds of being in the AN group compared to carers with no university education. The longer the time since dementia diagnosis, the less likely carers were to be in the AN group (Table S9).

The kernel PSM outperformed nearest neighbour and calliper (with a radius of 0.2) PSM in terms of average standardised difference of the covariates (Table S10). Kernel PSM performed well in terms of standardised difference in covariates (Figure S2) and matching of the propensity score across AN and non‐AN carers (Figure S3). Table 3 shows that using PSM to analyse carer outcomes produced results mostly in line with the linear regression results. The self‐efficacy measure related to service use was the only exception. AN carers were associated with greater self‐efficacy on service use by almost three points compared to non‐AN carers, although this association was statistically significant at the 5% level. The PSM produced a statistically insignificant estimate of the association between being an AN carer and costs, similar to that of the regression analysis. These results were robust to a more parsimonious specification of the logit regression for the estimation of the propensity score (Table S11).

### 3.6 IV analysis

Travel time to the closest AN provider is on average 13 min and its distribution is right‐skewed (Figure S4). Non‐AN carers were 17 min (0.286 hr) on average away from AN services whilst AN carers were 9 min away (0.151 hr). This difference is statistically significant at the 1% level. Travel time was a strong instrument as the Cragg‐Donald *F* statistic is between 41 and 56 (Table S12). We could not reject the hypothesis of no effect of travel time on outcomes when additional instruments (i.e. type of local authority dummies) are employed. This suggested that travel time has no relationship with the outcomes and was therefore a suitable variable to use in this part of our analysis.

Finally, Table 3 shows the results of the IV approach for outcomes and costs when travel time was used as an instrument. IV results were in line with those from the regression and PSM analysis showing a statistically insignificant effect of AN on outcomes and costs, except for ASCOT‐Carer which is weakly significant (at the 10% level). When standard error in the IV regressions were clustered within local authorities the effect of AN on happiness yesterday became statistically significant at the 5% level (Table S5). Results remain robust to a specification including fewer covariates (Table S13).

## 4 DISCUSSION AND CONCLUSIONS

Carers of people with dementia receiving AN services reported having slightly lower CRQoL and subjective well‐being than carers of people with dementia not receiving AN services. After controlling for differences in their observed and unobserved characteristics, however, we found that the CRQoL, self‐efficacy and subjective well‐being of AN carers was similar if not better than carers without access to AN services. The costs of health and social care services were similar across the two groups.

To our knowledge this is the first time that outcomes and costs have been compared in AN versus non‐AN carers. Our analysis used data from a recent feasibility survey (2017). We received 346 completed questionnaires which were in scope and most questionnaires were answered fully. The size and extent of these data allowed us to use different econometric techniques in an attempt to control for any systematic differences in the characteristics of AN and non‐AN carers, thereby minimising the risk of bias due to confounding.

Our study, however, has several limitations. We cannot be sure that our non‐probability sample reflects a fully representative sample of the target population of carers of people with dementia and this impacts on the transferability of our results beyond our sample of carers.

Our analysis is based on cross‐sectional, observational data. Although we controlled for several observed confounders, there exists a risk of confounding if AN and non‐AN carers are systematically different in characteristics that determine their outcomes and costs, and that we are not able to control for. The IV analysis helps us to address the selection bias due to unobserved characteristics and it relies on our choice of instrument and refers to the subgroup of carers who were induced to take up the AN service given their proximity to the service.

The data we used were collected at a single point in time. The analysis of both outcomes and costs rests on two important assumptions: (a) differences in observed and unobserved characteristics between carers in the treatment and control group remain similar over time in both pre‐ and post‐intervention hypothetical periods; and (b) the effect of AN is constant over time. The cross‐sectional survey design, however, allowed us to undertake this study with moderate uncertainty by reducing the demand on carers' time. Future studies may build upon our experience by implementing the survey questionnaire over multiple time points.

Admiral Nursing is a complex intervention and it is not possible to fully disentangle the effect on carers who received AN support in the past from other support services which they may also have utilised. Diversity in the referral process, such as referral to AN after a triage assessment or via self‐referral across AN providers, may generate high heterogeneity within the group of AN carers, hampering us from identifying an effect.

A feature of the survey design was that questions on service use related to the past 4 weeks for those services we thought would be used on a regular basis. In this way, we aimed to reduce recall bias. Moreover, the use of a postal, self‐administered survey meant that we could not comprehensively measure level of dementia severity, which is likely to affect the carer's needs. We could not find an appropriate measure in the published literature, hence using our own, low carer burden questionnaire which provides only partial information. Table S4 shows that, in the regression analysis, higher levels of perceived severity are always statistically significantly associated with worse outcomes.

We were unable to include an ASCOT‐Carer score which included preference weights for the UK population as this is not yet available (Batchelder et al., 2017). As an interim approach, we summed the domains and this assumes that all the domains are equally important. Finally, we put a monetary value or cost on informal care time using the proxy good method. This might involve an overestimation and it is only one out of many approaches to value informal care (Van den Berg, Brouwer, & Koopmanschap, 2004; Weatherly et al., 2014).

Considering these limitations, our findings provide an initial indication as to whether AN services could offer value for money. Full‐scale evaluation is required to make more definitive recommendations. Future research could build on our survey and collect data over multiple time‐points to better estimate the causal effect of AN services on carer outcomes and costs. Our survey could also be adapted to explore the outcomes and costs of other services for carers of people with dementia.
