# Supplementary material for: Investigating the economic case of a service to support carers of people with dementia: A cross‐sectional survey‐based feasibility study in England
Source: Health Soc Care Community. 2019 Jun 21;27(5):e734–43. doi: 10.1111/hsc.12799 (PMC6771746; doi:10.1111/hsc.12799)

**Supporting Material**

Table S1 – Unit costs of health and social care resources.

| **Item** | **Unit cost, £** | **Source** | **Notes** |
| --- | --- | --- | --- |
| **Hospital services** | | | |
| Hospital outpatient appointment | 120 | NHS reference costs 2016 | Average consultant led, non-consultant led and outpatient procedures. |
| Planned hospital admission without staying overnight | 733 | NHS reference costs 2016 | Average day case |
| Planned hospital admission with an overnight stay | 3750 | NHS reference costs 2016 | Average elective inpatient |
| Unplanned hospital admission | 1609 | NHS reference costs 2016 | Trim point=  Average non-elective inpatient, including short stay |
| Other hospital admissions | 389 | NHS reference costs 2016 | Regular day or night admissions |
| **Other health care services (rounded to units)** | | | |
| GP appointment | 36 | PSSRU 2016 p.154 | Per surgery consultation of 9.22 minutes including direct care staff costs and qualification costs |
| Nurse appointment | 11 | PSSRU 2016 p.152  PSSRU 2015 p.183 | Nurse based at GP practice including qualification costs: £44 per hour  Average contact duration is 15.5 minutes (PSSRU 2015) |
| Nurse specialist appointment | 13 | PSSRU 2016 p.151  PSSRU 2015 p.184 | Nurse Band 7 including qualification costs: £52 per hour  Assumes Average contact duration is 15.5 minutes (PSSRU 2015) as per nurse based at GP practice. |
| Therapist appointment: occupational therapist, physiotherapist, speech therapist, chiropodist, podiatrist | 9 | PSSRU 2016 p.194 | Allied health professionals Band 5: £34 per hour  Assumes Average contact duration is 15.5 minutes (PSSRU 2015) as per nurse based at GP practice. |
| **Social care services** | | | |
| Home care, per appointment | 12 | PSSRU 2016 p.169 | Face-to-face weekday: £24 per hour  Assume 30 minutes per session (ref UKHCA 2016 report) |
| Day care, per hour | 61 | PSSRU 2016 p.37 | £61 per client attendance |
| Meals, per meal | 4 | Glendenning *et al.* p.201; inflated to 2015-16 |  |
| Social service appointment: social worker | 20 | PSSRU 2016 p.165 | Per hour of client-related work including qualifications=£79  Assumes Average contact duration is 15.5 minutes (PSSRU 2015) as per nurse based at GP practice. |
| Memory café, per session | 14 | Rotary club 2015 | Based on example of memory café, at £138.33/monthly session. Assumes 10 carer-dementia pairs per session. |

Table S2 – Unit costs used for costing informal care with proxy good method.

| **Item** | **Unit cost, £** | **Source** | **Notes** | **Informal care activity** |
| --- | --- | --- | --- | --- |
| Paid carer, per hour | 24.6 | PSSRU 2016 p.169 | Weighted average of weekday, night and weekend hours. | Personal care, physical help, keeping company, transport, keeping an eye |
| Citizen Advice Bureau adviser, per hour | 52 | PSSRU 2016 p.171 | Knapp *et al*., 2013 used the cost of a family support worker to reflect the cost of an adviser. | Dealing with care services and benefits |
| Finance administrator, per hour | 25 | PSSRU 2016 p.146; NHS Agenda for change | Finance officer is at Agenda for change Band 3 ([here](https://www.healthcareers.nhs.uk/explore-roles/corporate-services/finance-staff)). | Dealing with other paperwork and financial matters |
| Cleaner/handy person, per hour | 21 | PSSRU 2016 p.146 | Housekeeping assistant is at Agenda for change Band 1. Mid-point Band 1 is £15,500/year. See [here](https://www.healthcareers.nhs.uk/about/careers-nhs/nhs-pay-and-benefits/agenda-change-pay-rates). Calculated from the ratio wages/hourly cost Band 2. | Practical help |

Table S3 – Descriptive statistics on Admiral Nursing dummy and observed confounders.

| Covariate | | All carers | | | |  | AN | | Non-AN | |
| --- | --- | --- | --- | --- | --- | --- | --- | --- | --- | --- |
|  |  | Freq | Mean | 95% CI | |  | Freq | Mean | Freq | Mean |
| Carer characteristics | Admiral nursing | 132 | 0.468 | 0.410 | 0.527 |  | 132 | 1.000 | 0 | 0.000 |
|  | Male (ref) | 87 | 0.309 | 0.257 | 0.365 |  | 45 | 0.341 | 42 | 0.280 |
|  | Female | 195 | 0.691 | 0.635 | 0.743 |  | 87 | 0.659 | 108 | 0.720 |
|  | 16 to 54 years old (ref) | 50 | 0.177 | 0.137 | 0.227 |  | 16 | 0.121 | 34 | 0.227 |
|  | 55 to 64 years old | 71 | 0.252 | 0.204 | 0.306 |  | 29 | 0.220 | 42 | 0.280 |
|  | 65 to 69 years old | 31 | 0.110 | 0.078 | 0.152 |  | 17 | 0.129 | 14 | 0.093 |
|  | 70 to 74 years old | 42 | 0.149 | 0.112 | 0.196 |  | 22 | 0.167 | 20 | 0.133 |
|  | 75 years or older | 88 | 0.312 | 0.260 | 0.369 |  | 48 | 0.364 | 40 | 0.267 |
|  | Below university education (ref) | 203 | 0.720 | 0.664 | 0.769 |  | 102 | 0.773 | 101 | 0.673 |
|  | Bachelor's degree | 55 | 0.195 | 0.153 | 0.246 |  | 26 | 0.197 | 29 | 0.193 |
|  | Master's degree and above | 24 | 0.085 | 0.058 | 0.124 |  | 4 | 0.030 | 20 | 0.133 |
|  | Full/part-time job | 59 | 0.209 | 0.165 | 0.261 |  | 21 | 0.159 | 38 | 0.253 |
|  | Look after full time | 70 | 0.248 | 0.201 | 0.302 |  | 39 | 0.295 | 31 | 0.207 |
|  | Retired | 164 | 0.582 | 0.523 | 0.638 |  | 81 | 0.614 | 83 | 0.553 |
|  | No financial difficulties (ref) | 66 | 0.234 | 0.188 | 0.287 |  | 26 | 0.197 | 40 | 0.267 |
|  | Some financial difficulties | 92 | 0.326 | 0.274 | 0.383 |  | 41 | 0.311 | 51 | 0.340 |
|  | Severe financial difficulties | 89 | 0.316 | 0.264 | 0.373 |  | 50 | 0.379 | 39 | 0.260 |
|  | Don't know/prefer not to say | 35 | 0.124 | 0.090 | 0.168 |  | 15 | 0.114 | 20 | 0.133 |
|  | EQ-5D score | 282 | 0.774 | 0.752 | 0.795 |  | 132 | 0.752 | 150 | 0.793 |
|  | Main carer (ref) | 261 | 0.926 | 0.888 | 0.951 |  | 129 | 0.977 | 132 | 0.880 |
|  | Joint or not main carer | 21 | 0.075 | 0.049 | 0.112 |  | 3 | 0.023 | 18 | 0.120 |
|  | Care recipient's spouse/partner (ref) | 190 | 0.674 | 0.617 | 0.726 |  | 98 | 0.742 | 92 | 0.613 |
|  | Care recipient's child/child-in-law | 81 | 0.287 | 0.237 | 0.343 |  | 30 | 0.227 | 51 | 0.340 |
|  | Other relationship with care recipient | 11 | 0.039 | 0.022 | 0.069 |  | 4 | 0.030 | 7 | 0.047 |
|  | Personal care | 198 | 0.702 | 0.646 | 0.753 |  | 99 | 0.750 | 99 | 0.660 |
|  | Physical care | 136 | 0.482 | 0.424 | 0.541 |  | 71 | 0.538 | 65 | 0.433 |
|  | Total hours of care | 282 | 12 | 11 | 13 |  | 132 | 13 | 150 | 11 |
|  | Caring for less than 1 year (ref) | 18 | 0.064 | 0.040 | 0.099 |  | 6 | 0.046 | 12 | 0.080 |
|  | Caring for 1 to 3 years | 91 | 0.323 | 0.270 | 0.380 |  | 48 | 0.364 | 43 | 0.287 |
|  | Caring for 3 to 5 years | 78 | 0.277 | 0.227 | 0.332 |  | 31 | 0.235 | 47 | 0.313 |
|  | Caring for 5 to 10 years | 66 | 0.234 | 0.188 | 0.287 |  | 33 | 0.250 | 33 | 0.220 |
|  | Caring for 10 years or more | 29 | 0.103 | 0.072 | 0.144 |  | 14 | 0.106 | 15 | 0.100 |
|  | Replacement for a break (ref) | 116 | 0.411 | 0.355 | 0.470 |  | 43 | 0.326 | 73 | 0.487 |
|  | No replacement for a break | 166 | 0.589 | 0.530 | 0.645 |  | 89 | 0.674 | 77 | 0.513 |
| Care recipient characteristics | 45 to 64 years old (ref) | 28 | 0.099 | 0.069 | 0.140 |  | 10 | 0.076 | 18 | 0.120 |
|  | 65 to 74 years old | 57 | 0.202 | 0.159 | 0.253 |  | 28 | 0.212 | 29 | 0.193 |
|  | 75 years or older old | 197 | 0.699 | 0.642 | 0.750 |  | 94 | 0.712 | 103 | 0.687 |
|  | Duration of symptoms under 1 year (ref) | 13 | 0.046 | 0.027 | 0.078 |  | 8 | 0.061 | 5 | 0.033 |
|  | Duration of symptoms from 1 to 5 years | 166 | 0.589 | 0.530 | 0.645 |  | 79 | 0.598 | 87 | 0.580 |
|  | Duration of symptoms from 6 to 10 years | 79 | 0.280 | 0.231 | 0.336 |  | 35 | 0.265 | 44 | 0.293 |
|  | Duration of symptoms for 11 years or more | 24 | 0.085 | 0.058 | 0.124 |  | 10 | 0.076 | 14 | 0.093 |
|  | Formal diagnosis of dementia (ref) | 272 | 0.965 | 0.935 | 0.981 |  | 128 | 0.970 | 144 | 0.960 |
|  | No diagnosis of dementia/don't know | 10 | 0.036 | 0.019 | 0.065 |  | 4 | 0.030 | 6 | 0.040 |
|  | Alzheimer's | 171 | 0.606 | 0.548 | 0.662 |  | 68 | 0.515 | 103 | 0.687 |
|  | Vascular dementia | 94 | 0.333 | 0.280 | 0.391 |  | 58 | 0.439 | 36 | 0.240 |
|  | Other dementia | 76 | 0.270 | 0.221 | 0.325 |  | 34 | 0.258 | 42 | 0.280 |
|  | Mild perceived severity (ref) | 29 | 0.103 | 0.072 | 0.144 |  | 9 | 0.068 | 20 | 0.133 |
|  | Moderate perceived severity | 178 | 0.631 | 0.573 | 0.686 |  | 85 | 0.644 | 93 | 0.620 |
|  | Severe perceived severity | 75 | 0.266 | 0.217 | 0.321 |  | 38 | 0.288 | 37 | 0.247 |
| Obs=total number of observations; Freq=number of observations for each category (it is equivalent to Obs for continuous variables); Mean=proportion for dummy variables and average for continuous variables; CI=confidence intervals; ref=reference category. | | | | | | | | | | |
| The sample included 282 carers, of which 132 AN carers and 150 non-AN carers. The descriptive statistics reported in this table were calculated on the ASCOT-Carer score's sample. | | | | | | | | | | |

Table S4 – Full results of the regression analysis on outcomes.

| Variable | | ASCOT-Carer score | Self-efficacy on symptoms management | Self-efficacy on service use | Overall life satisfaction | Happiness yesterday |
| --- | --- | --- | --- | --- | --- | --- |
|  | Admiral nursing | 0.382 | 1.243 | 1.990* | 0.087 | 0.433 |
|  |  | (0.397) | (1.317) | (1.060) | (0.266) | (0.279) |
| Carer characteristics | Female | -0.539 | -2.384 | -3.842*** | -0.166 | 0.001 |
|  |  | (0.495) | (1.446) | (1.232) | (0.334) | (0.344) |
|  | 55 to 64 years old | -0.134 | -2.399 | -1.712 | -0.387 | -0.618 |
|  |  | (0.705) | (1.982) | (1.853) | (0.439) | (0.466) |
|  | 65 to 69 years old | 0.127 | -2.214 | 0.596 | -0.181 | -0.300 |
|  |  | (0.979) | (3.030) | (2.805) | (0.664) | (0.719) |
|  | 70 to 74 years old | 0.202 | -1.761 | 1.448 | -0.015 | 0.227 |
|  |  | (0.903) | (2.777) | (2.510) | (0.641) | (0.654) |
|  | 75 years old or older | 0.833 | -3.149 | -5.370* | 0.629 | 0.545 |
|  |  | (1.023) | (3.090) | (2.796) | (0.721) | (0.735) |
|  | Bachelor's degree | -0.483 | -2.408* | -2.403* | -0.456 | -0.467 |
|  |  | (0.444) | (1.441) | (1.293) | (0.300) | (0.313) |
|  | Master's degree and above | -0.871 | -3.096 | -1.205 | -0.083 | 0.157 |
|  |  | (0.685) | (2.494) | (1.849) | (0.508) | (0.540) |
|  | Full/part-time job | 1.212* | 2.045 | 1.227 | 0.069 | -0.042 |
|  |  | (0.663) | (2.039) | (1.886) | (0.401) | (0.430) |
|  | Look after full time | 0.273 | 0.264 | 0.743 | 0.050 | 0.055 |
|  |  | (0.486) | (1.534) | (1.397) | (0.319) | (0.346) |
|  | Retired | 1.159** | 2.216 | 2.152 | 0.289 | 0.499 |
|  |  | (0.564) | (1.893) | (1.643) | (0.376) | (0.403) |
|  | Some financial difficulties | -1.531*** | -1.384 | -3.928*** | -0.830** | -1.075*** |
|  |  | (0.580) | (1.802) | (1.419) | (0.378) | (0.389) |
|  | Severe financial difficulties | -2.281*** | -2.936 | -3.926*** | -1.308*** | -1.382*** |
|  |  | (0.605) | (1.800) | (1.399) | (0.393) | (0.403) |
|  | Don't know/prefer not to say | -2.636*** | -1.904 | -4.371** | -0.740 | -1.136** |
|  |  | (0.782) | (2.338) | (1.837) | (0.472) | (0.490) |
|  | EQ-5D score | 7.131*** | 13.295*** | 8.581*** | 4.129*** | 4.160*** |
|  |  | (1.145) | (3.722) | (2.887) | (0.934) | (0.957) |
|  | Joint or not main carer | 0.543 | 2.790 | 3.307 | 0.823* | 0.535 |
|  |  | (0.962) | (1.898) | (2.112) | (0.483) | (0.484) |
|  | Care recipient's child/child-in-law | 0.889 | 0.375 | -2.327 | 0.336 | 0.407 |
|  |  | (0.818) | (2.534) | (2.211) | (0.570) | (0.603) |
|  | Other relationship with care recipient | 0.063 | -4.703 | -5.352* | -0.200 | -0.206 |
|  |  | (1.262) | (3.610) | (3.094) | (0.806) | (0.720) |
|  | Personal care | -0.495 | -1.076 | -0.896 | -0.214 | -0.031 |
|  |  | (0.505) | (1.553) | (1.294) | (0.314) | (0.350) |
|  | Physical care | -0.578 | 2.578* | 0.964 | 0.108 | 0.003 |
|  |  | (0.468) | (1.436) | (1.201) | (0.319) | (0.344) |
|  | Total hours of care | -0.084*** | 0.213** | 0.042 | -0.023 | -0.029 |
|  |  | (0.030) | (0.094) | (0.081) | (0.018) | (0.019) |
|  | Caring for 1 to 3 years | -0.142 | 2.512 | 6.458*** | 0.274 | 0.765 |
|  |  | (1.082) | (2.976) | (2.160) | (0.719) | (0.669) |
|  | Caring for 3 to 5 years | -0.195 | 1.496 | 4.667** | 0.331 | 0.895 |
|  |  | (1.142) | (3.174) | (2.213) | (0.742) | (0.710) |
|  | Caring for 5 to 10 years | -0.108 | 2.149 | 5.895** | -0.141 | 0.453 |
|  |  | (1.211) | (3.386) | (2.397) | (0.771) | (0.774) |
|  | Caring for 10 years or more | -0.658 | 0.493 | 4.568 | -0.021 | 0.360 |
|  |  | (1.342) | (4.155) | (3.045) | (0.845) | (0.892) |
|  | No replacement for a break | -1.192*** | -3.433** | -2.829*** | -0.746*** | -0.772*** |
|  |  | (0.440) | (1.341) | (1.083) | (0.281) | (0.293) |
| Care recipient characteristics | 65 to 74 years old | 0.782 | 0.187 | 1.552 | 0.529 | 0.640 |
|  |  | (0.708) | (2.736) | (2.078) | (0.507) | (0.520) |
|  | 75 years old or older | 0.665 | 2.396 | 4.932** | 0.290 | 0.367 |
|  |  | (0.762) | (2.884) | (2.095) | (0.575) | (0.563) |
|  | Symptoms for 1 to 5 years | 0.107 | 1.565 | 0.402 | 0.961 | -0.185 |
|  |  | (1.175) | (3.644) | (3.661) | (0.760) | (0.635) |
|  | Symptoms for 6 to 10 years | -0.247 | 2.725 | 0.117 | 1.260 | 0.429 |
|  |  | (1.289) | (4.091) | (4.004) | (0.861) | (0.798) |
|  | Symptoms for 11 years or more | 0.993 | 6.705 | 1.174 | 1.567* | 0.985 |
|  |  | (1.450) | (4.809) | (4.508) | (0.910) | (0.953) |
|  | No diagnosis of dementia/don't know | -1.424 | -6.734*** | -4.891* | -0.208 | -0.329 |
|  |  | (0.926) | (2.574) | (2.584) | (0.454) | (0.486) |
|  | Vascular dementia | -0.722 | -1.211 | -1.682 | -0.096 | 0.159 |
|  |  | (0.445) | (1.373) | (1.148) | (0.308) | (0.326) |
|  | Other dementia | -0.561 | -0.827 | -4.162*** | -0.062 | 0.218 |
|  |  | (0.450) | (1.618) | (1.308) | (0.322) | (0.338) |
|  | Moderate perceived severity | -0.585 | -2.828 | -3.692** | -0.827* | -1.012* |
|  |  | (0.715) | (2.080) | (1.688) | (0.474) | (0.531) |
|  | Severe perceived severity | -1.722** | -5.450** | -4.208** | -1.574*** | -1.795*** |
|  |  | (0.783) | (2.533) | (2.066) | (0.552) | (0.597) |
|  | Constant | 7.928*** | 17.986*** | 18.970*** | 2.271 | 2.782* |
|  |  | (2.007) | (5.858) | (4.716) | (1.435) | (1.482) |
| Observations | | 282 | 274 | 268 | 287 | 285 |
| R-squared | | 0.459 | 0.260 | 0.350 | 0.340 | 0.335 |
| Most of the coefficients on dummies are interpreted in relation to the reference category. The reference categories for carer characteristics are respectively: Male, Age 16 to 54 years, Below university education, No financial difficulties. The reference categories for the characteristics of the caring role are: Main carer status, Person with dementia is spouse/partner, Duration of caring under 1 year. Finally, the reference categories for the care recipient characteristics are: Age 45 to 64, Duration of symptoms under 1 year, Severity of dementia is Mild. Table 3 shows the reference categories. | | | | | | |
| Robust standard errors in parentheses; ***=p-value<0.01, **=p-value<0.05, *=p-value<0.1 | | | | | | |

Table S5 – Regression and IV results after clustering standard errors or using local authority random effects.

|  | ASCOT-Carer score | Self-efficacy on symptoms management | Self-efficacy on service use | Overall life satisfaction | Happiness yesterday |  | Overall costs | Carer's healthcare costs | Care recipient's healthcare costs | Social care costs |
| --- | --- | --- | --- | --- | --- | --- | --- | --- | --- | --- |
|  |  | | | | | | | | | |
|  | Regression with clustered standard errors within local authorities | | | | | | | | | |
| Coeff | 0.361 | 1.020 | 1.651 | 0.047 | 0.375 |  | 46.455 | -183.693 | -105.097 | 77.329 |
| Std Err | (0.357) | (1.454) | (1.286) | (0.318) | (0.363) |  | (193.965) | (182.582) | (139.727) | (105.901) |
|  |  |  |  |  |  |  |  |  |  |  |
|  | Regression with local authority random effects and clustered standard errors | | | | | | | | | |
| Coeff | 0.361 | 1.020 | 1.078 | 0.103 | 0.490 |  | 46.455 | -178.775 | -48.940 | 77.333 |
| Std Err | (0.333) | (1.353) | (1.370) | (0.343) | (0.397) |  | (177.772) | (151.431) | (225.510) | (98.388) |
|  |  |  |  |  |  |  |  |  |  |  |
|  | Instrumental Variables with clustered standard errors within local authorities | | | | | | | | | |
| Coeff | 1.472 | 2.899 | 2.586 | 0.174 | 0.937 |  | -29.413 | -44.331 | 187.689 | -440.286 |
| Std Err | (0.874)* | (2.651) | (2.772) | (0.461) | (0.454)** |  | (336.732) | (265.401) | (209.150) | (293.622) |
| Coeff=estimated coefficient on the Admiral Nursing dummy; Std Err=robust standard errors. | | | | | | | | | | |
| In all regressions, we controlled for carer characteristics (including gender, age, education, work situation, household financial difficulties, whether the carer was a sole carer, relationship with the care recipient, type and amount of time of care provided, number of years caring, availability of a replacement for a break, and HRQoL) and care recipient characteristics (including age, duration of symptoms of dementia, existence of a formal diagnosis, type of dementia such as Alzheimer, vascular dementia, or other type of dementia, and perceived severity of dementia). In the IV regression, the instrument was the travel time to the closest AN provider. | | | | | | | | | | |
| Clustered standard errors in parenthesis; ***=p-value<0.01, **=p-value<0.05, *=p-value<0.1 | | | | | | | | | | |

Table S6 – Regression results using GLM for outcomes and two-part model for costs.

|  | ASCOT-Carer score | Self-efficacy on symptoms management | Self-efficacy on service use | Overall life satisfaction | Happiness yesterday |  | Overall costs | Carer's healthcare costs | Care recipient's healthcare costs | Social care costs |
| --- | --- | --- | --- | --- | --- | --- | --- | --- | --- | --- |
|  |  | | | | |  | Two-part model (logit, GLM with log link and normal distribution) | | | |
|  | GLM regression with log link and normal distribution | | | | |  |  |  |  |  |
| Coeff | 0.168 | 0.836 | 1.569 | 0.078 | 0.295 |  | 192.415 | - | - | 266.575 |
| Std Err | (0.404) | (1.214) | (0.987) | (0.249) | (0.251) |  | (582.366) | - | - | (323.136) |
|  |  |  |  |  |  |  | Two-part model (logit, GLM with log link and gamma distribution) | | | |
|  | GLM regression with log link and gamma distribution | | | | |  |  |  |  |  |
| Coeff | 0.689 | 1.663 | 2.522 | 0.062 | 0.613 |  | 409.218 | -55.264 | -54.377 | 118.241 |
| Std Err | (0.425) | (1.401) | (1.175)** | (0.314) | (0.339)* |  | (252.277) | (79.839) | (93.319) | (151.750) |
| Coeff=estimated coefficient on the Admiral Nursing dummy; Std Err=robust standard errors. | | | | | | | | | | |
| The two-part model using GLM with log link and normal distribution in the second part did not converge when the dependent variable was the carer's healthcare costs and the care recipient's healthcare costs. Estimates are therefore omitted. | | | | | | | | | | |
| In all regressions, we controlled for carer characteristics (including gender, age, education, work situation, household financial difficulties, whether the carer was a sole carer, relationship with the care recipient, type and amount of time of care provided, number of years caring, availability of a replacement for a break, and HRQoL) and care recipient characteristics (including age, duration of symptoms of dementia, existence of a formal diagnosis, type of dementia such as Alzheimer, vascular dementia, or other type of dementia, and perceived severity of dementia). In the IV regression, the instrument was the travel time to the closest AN provider. | | | | | | | | | | |
| Robust standard errors in parenthesis; ***=p-value<0.01, **=p-value<0.05, *=p-value<0.1 | | | | | | | | | | |

Table S7 – Missing data.

| Variable | Resp | Miss | Prop |
| --- | --- | --- | --- |
| Outcome | | | |
| ASCOT-Carer score | 317 | 29 | 8.4% |
| Self-efficacy on symptoms management | 310 | 36 | 10.4% |
| Self-efficacy on service use | 302 | 44 | 12.7% |
| Overall life satisfaction | 330 | 16 | 4.6% |
| Happiness yesterday | 328 | 18 | 5.2% |
| Costs | | | |
| Overall costs | 260 | 86 | 24.9% |
| Carer's healthcare costs | 306 | 40 | 11.6% |
| Care recipient's healthcare costs | 297 | 49 | 14.2% |
| Social care costs | 307 | 39 | 11.3% |
| Carer characteristics | | | |
| Gender | 340 | 6 | 1.7% |
| Age | 340 | 6 | 1.7% |
| Education | 339 | 7 | 2.0% |
| Work situation | 346 | 0 | 0.0% |
| Household financial difficulties | 316 | 30 | 8.7% |
| EQ-5D score | 330 | 16 | 4.6% |
| Caring status | 336 | 10 | 2.9% |
| Relationship with the care recipient | 333 | 13 | 3.8% |
| Provision of personal care | 346 | 0 | 0.0% |
| Provision of physical care | 346 | 0 | 0.0% |
| Total hours of caring | 328 | 18 | 5.2% |
| Duration of caring | 339 | 7 | 2.0% |
| Availability of a replacement for a break | 337 | 9 | 2.6% |
| Care recipient characteristics | | | |
| Age | 331 | 15 | 4.3% |
| Duration of symptoms of dementia | 339 | 7 | 2.0% |
| Formal diagnosis of dementia | 340 | 6 | 1.7% |
| Type of dementia | 346 | 0 | 0.0% |
| Perceived severity of dementia | 339 | 7 | 2.0% |
| Instruments | | | |
| Travel time | 327 | 19 | 5.5% |
| Unitary LA | 327 | 19 | 5.5% |
| County LA | 327 | 19 | 5.5% |
| London LA | 327 | 19 | 5.5% |
| Metropolitan LA | 327 | 19 | 5.5% |
| Resp=number of respondents, Miss=Number of missing responses, Prop=proportion of missing responses. | | | |
| Numbers and proportions are calculated considering the 346 in-scope questionnaires. | | | |

Table S8 – Regression results using fewer covariates.

| Variable | | ASCOT-Carer score | Self-efficacy on symptoms management | Self-efficacy on service use | Overall life satisfaction | Happiness yesterday |  | Overall costs | Carer's healthcare costs | Care recipient's healthcare costs | Social care costs |
| --- | --- | --- | --- | --- | --- | --- | --- | --- | --- | --- | --- |
|  | Admiral nursing | 0.290 | 0.986 | 2.334** | 0.008 | 0.414 |  | 59.667 | -149.215 | -90.373 | 62.143 |
|  |  | (0.381) | (1.273) | (1.033) | (0.258) | (0.270) |  | (194.057) | (125.449) | (129.852) | (143.216) |
| Carer characteristics | Female | -0.565 | -2.293 | -4.517*** | -0.242 | -0.128 |  | -30.147 | 202.421 | -1.196 | 9.332 |
|  |  | (0.478) | (1.449) | (1.248) | (0.316) | (0.320) |  | (207.972) | (183.805) | (145.176) | (169.158) |
|  | 75 years old or older | 0.366 | -0.739 | -3.738*** | 0.658* | 0.562 |  | -25.753 | 219.301 | -4.414 | 29.910 |
|  |  | (0.524) | (1.574) | (1.281) | (0.341) | (0.347) |  | (245.002) | (192.749) | (174.937) | (172.547) |
|  | Bachelor's degree | -0.543 | -2.193 | -2.523** | -0.431 | -0.460 |  | 395.924* | -125.268* | -101.558 | 377.812** |
|  |  | (0.459) | (1.399) | (1.244) | (0.292) | (0.305) |  | (217.122) | (68.130) | (99.827) | (156.532) |
|  | Master's degree and above | -0.925 | -3.516 | -2.270 | -0.138 | 0.038 |  | 383.993 | -35.129 | -129.931 | 314.823 |
|  |  | (0.661) | (2.356) | (1.869) | (0.481) | (0.510) |  | (376.738) | (270.650) | (187.357) | (257.183) |
|  | Retired | 0.453 | 0.768 | 2.513** | 0.211 | 0.530* |  | 209.308 | 211.766 | 19.017 | 242.305* |
|  |  | (0.447) | (1.427) | (1.237) | (0.293) | (0.309) |  | (214.228) | (145.108) | (141.940) | (142.832) |
|  | Some financial difficulties | -1.595*** | -1.059 | -3.342** | -0.737** | -1.006*** |  | 25.096 | -10.190 | 9.388 | -27.000 |
|  |  | (0.551) | (1.719) | (1.372) | (0.355) | (0.360) |  | (207.259) | (89.092) | (120.851) | (149.520) |
|  | Severe financial difficulties | -2.319*** | -2.207 | -3.713*** | -1.287*** | -1.379*** |  | 55.588 | 155.485 | -87.986 | 270.256 |
|  |  | (0.563) | (1.709) | (1.378) | (0.365) | (0.376) |  | (204.679) | (154.623) | (119.780) | (174.600) |
|  | Prefer not to say financial difficulties | -2.857*** | -1.498 | -4.474** | -0.792* | -1.200** |  | 733.524 | 396.569 | 416.838 | 219.481 |
|  |  | (0.773) | (2.254) | (1.838) | (0.444) | (0.481) |  | (458.802) | (293.583) | (324.569) | (240.939) |
|  | EQ-5D score | 7.096*** | 14.795*** | 9.571*** | 4.174*** | 4.169*** |  | 426.484 | -448.593 | -69.056 | 398.446 |
|  |  | (1.076) | (3.652) | (2.527) | (0.918) | (0.946) |  | (413.041) | (458.009) | (266.576) | (336.976) |
|  | Joint or not main carer | 0.694 | 4.135** | 3.000 | 1.026** | 0.797* |  | 292.988 | -172.807 | 120.858 | 186.536 |
|  |  | (0.904) | (1.708) | (2.074) | (0.452) | (0.478) |  | (481.382) | (139.352) | (320.606) | (252.849) |
|  | Total hours of care | -0.102*** | 0.235*** | 0.082 | -0.023 | -0.027 |  | 10.482 | -11.903* | 13.182 | -10.776 |
|  |  | (0.028) | (0.087) | (0.077) | (0.017) | (0.017) |  | (13.672) | (6.296) | (9.195) | (9.575) |
|  | Caring for 5 years or more | -0.459 | 0.355 | 0.257 | -0.234 | -0.043 |  | -171.311 | -155.215 | -395.307** | 309.536* |
|  |  | (0.432) | (1.429) | (1.094) | (0.269) | (0.304) |  | (223.739) | (104.922) | (154.709) | (167.524) |
|  | No replacement for a break | -1.275*** | -3.091** | -2.469** | -0.686** | -0.700** |  | 27.120 | -248.147* | -95.026 | 52.929 |
|  |  | (0.427) | (1.262) | (1.055) | (0.267) | (0.276) |  | (191.521) | (135.473) | (134.575) | (143.035) |
| Care recipient characteristics | 75 years old or older | 0.418 | 2.560* | 2.516** | 0.067 | 0.065 |  | 161.804 | 34.627 | 198.617 | -53.246 |
|  |  | (0.458) | (1.540) | (1.229) | (0.306) | (0.331) |  | (204.810) | (80.891) | (137.661) | (143.388) |
|  | Symptoms for 11 years or more | 0.811 | 4.123* | 0.574 | 0.511 | 0.726 |  | 321.109 | -80.269 | 360.467 | -111.081 |
|  |  | (0.632) | (2.227) | (1.912) | (0.405) | (0.553) |  | (419.557) | (244.166) | (321.130) | (271.786) |
|  | No diagnosis of dementia/don't know | -1.729* | -7.323*** | -5.335** | -0.103 | -0.124 |  | 270.059 | -190.345 | 162.840 | -209.160 |
|  |  | (0.953) | (2.323) | (2.601) | (0.441) | (0.510) |  | (647.331) | (230.279) | (588.173) | (312.728) |
|  | Vascular dementia | -0.733* | -0.972 | -1.369 | -0.048 | 0.148 |  | -2.642 | 43.662 | 251.909** | -183.553 |
|  |  | (0.425) | (1.320) | (1.158) | (0.288) | (0.299) |  | (188.934) | (113.589) | (116.078) | (144.196) |
|  | Other dementia | -0.606 | -0.686 | -3.824*** | -0.075 | 0.148 |  | 20.163 | 147.075 | 235.617 | -102.371 |
|  |  | (0.442) | (1.545) | (1.211) | (0.321) | (0.336) |  | (235.290) | (175.882) | (177.272) | (175.228) |
|  | Moderate perceived severity | -0.570 | -2.420 | -3.591** | -0.548 | -0.813* |  | -181.598 | 57.881 | -147.063 | 19.629 |
|  |  | (0.641) | (1.838) | (1.539) | (0.401) | (0.431) |  | (301.172) | (111.574) | (197.803) | (157.864) |
|  | Severe perceived severity | -1.930*** | -4.637** | -4.218** | -1.271*** | -1.541*** |  | 546.147 | 477.513* | 95.133 | 532.823** |
|  |  | (0.718) | (2.231) | (1.880) | (0.482) | (0.515) |  | (376.130) | (259.581) | (254.283) | (211.172) |
|  | Constant | 9.246*** | 18.735*** | 23.400*** | 3.408*** | 3.548*** |  | 68.503 | 401.477 | 204.128 | -115.178 |
|  |  | (1.473) | (4.486) | (3.315) | (1.136) | (1.188) |  | (570.341) | (569.224) | (394.070) | (394.955) |
| Observations | | 282 | 274 | 268 | 287 | 285 |  | 227 | 269 | 259 | 266 |
| R-squared | | 0.428 | 0.222 | 0.289 | 0.320 | 0.303 |  | 0.134 | 0.147 | 0.123 | 0.134 |
| Most of the coefficients on dummies are interpreted in relation to the reference category. The reference categories for carer characteristics are respectively: Male, Younger than 75 years old, Below university education, No financial difficulties, Main carer status, Duration of caring under 5 years. The reference categories for the care recipient characteristics are: Younger than 74 years old, Duration of symptoms under 11 years, Mild severity of dementia. | | | | | | | | | | | |
| Robust standard errors in parentheses; ***=p-value<0.01, **=p-value<0.05, *=p-value<0.1 | | | | | | | | | | | |

Table S9 – Logit regression on the Admiral Nursing dummy.

| Variable | | Admiral Nursing |
| --- | --- | --- |
| Carer's characteristics | Female | 0.816 |
|  | 55 to 64 years old | 1.905 |
|  | 65 to 69 years old | 3.129 |
|  | 70 to 74 years old | 2.036 |
|  | 75 years old or older | 1.935 |
|  | Bachelor's degree | 1.015 |
|  | Master's degree and above | 0.222** |
|  | Full/part-time job | 1.115 |
|  | Look after full time | 1.241 |
|  | Retired | 0.832 |
|  | Some financial difficulties | 1.088 |
|  | Severe financial difficulties | 1.697 |
|  | Don't know/prefer not to say | 1.258 |
|  | EQ-5D score | 0.483 |
| Caring role | Joint or not main carer | 0.398 |
|  | Child/child-in-law | 0.829 |
|  | Other relationship | 1.154 |
|  | Personal care | 1.300 |
|  | Physical care | 1.117 |
|  | Total hours of care | 1.009 |
|  | Caring for 1 to 3 years | 3.834* |
|  | Caring for 3 to 5 years | 2.327 |
|  | Caring for 5 to 10 years | 3.860 |
|  | Caring for 10 years or more | 3.871 |
|  | No replacement for a break | 1.512 |
| Care recipient's characteristics | 65 to 74 years old | 0.986 |
|  | 75 years old or older | 1.132 |
|  | Symptoms for 1 to 5 years | 0.135** |
|  | Symptoms for 6 to 10 years | 0.084** |
|  | Symptoms for 11 years or more | 0.067** |
|  | No diagnosis/don't know | 0.668 |
|  | Vascular dementia | 2.456*** |
|  | Other dementia | 1.055 |
|  | Moderate severity | 2.367 |
|  | Severe severity | 2.444 |
|  | Constant | 0.440 |
| Observations | | 282 |
| Logit regression analysis on ASCOT-Carer score's sample. The Admiral Nursing dummy is the dependent variable. Most of the coefficients on dummies are interpreted in relation to the reference category. Table 3 shows the reference categories. | | |
| Robust standard errors in parentheses; *** p<0.01, ** p<0.05, * p<0.1. | | |

Table S10 – Comparison between PSM techniques on average standardised difference of the covariates.

| Technique | Sample | Mean bias | Median bias |
| --- | --- | --- | --- |
| Kernel | Unmatched | 14.4 | 11.4 |
|  | Matched | 3.4 | 3.1 |
| Nearest neighbour | Unmatched | 14.4 | 11.4 |
|  | Matched | 8.4 | 7.6 |
| Calliper (0.2) | Unmatched | 14.4 | 11.4 |
|  | Matched | 8.4 | 7.6 |

Table S11 – PSM results when the logit regression uses fewer covariates.

| Variable | | ASCOT-Carer score | Self-efficacy on symptoms management | Self-efficacy on service use | Overall life satisfaction | Happiness yesterday |  | Overall costs | Carer's healthcare costs | Care recipient's healthcare costs | Social care costs |
| --- | --- | --- | --- | --- | --- | --- | --- | --- | --- | --- | --- |
|  | Admiral nursing | 0.538 | 1.474 | 2.937 | 0.133 | 0.541 |  | -43.233 | -175.572 | -130.078 | -12.104 |
|  |  | (0.527) | (1.471) | (1.288)** | (0.314) | (0.330) |  | (211.553) | (129.353) | (132.273) | (137.414) |
| Estimated coefficients from Logit regression in which the AN dummy is the dependent variable | | | | | | | | | | | |
| Carer characteristics | Female | -0.296 | -0.185 | -0.234 | -0.236 | -0.211 |  | -0.419 | -0.256 | -0.168 | -0.367 |
|  |  | (0.322) | (0.327) | (0.326) | (0.317) | (0.318) |  | (0.361) | (0.327) | (0.327) | (0.338) |
|  | 75 years old or older | 0.203 | 0.376 | 0.287 | 0.337 | 0.322 |  | -0.059 | 0.088 | 0.288 | 0.104 |
|  |  | (0.355) | (0.366) | (0.366) | (0.355) | (0.356) |  | (0.415) | (0.364) | (0.374) | (0.369) |
|  | Bachelor's degree | -0.030 | 0.042 | -0.028 | 0.002 | -0.023 |  | 0.119 | -0.045 | 0.095 | -0.009 |
|  |  | (0.334) | (0.338) | (0.339) | (0.333) | (0.333) |  | (0.386) | (0.342) | (0.361) | (0.340) |
|  | Master's degree and above | -1.660 | -1.944 | -1.953 | -1.603 | -1.623 |  | -2.150 | -1.481 | -1.515 | -1.933 |
|  |  | (0.621)*** | (0.690)*** | (0.685)*** | (0.620)*** | (0.618)*** |  | (0.848)** | (0.633)** | (0.629)** | (0.708)*** |
|  | Retired | -0.047 | -0.016 | 0.040 | -0.063 | -0.062 |  | -0.001 | 0.068 | -0.059 | -0.049 |
|  |  | (0.315) | (0.324) | (0.327) | (0.316) | (0.316) |  | (0.379) | (0.320) | (0.333) | (0.332) |
|  | Some financial difficulties | 0.104 | 0.154 | 0.151 | 0.086 | 0.079 |  | 0.392 | 0.015 | 0.111 | 0.325 |
|  |  | (0.364) | (0.373) | (0.372) | (0.360) | (0.361) |  | (0.413) | (0.368) | (0.374) | (0.379) |
|  | Severe financial difficulties | 0.446 | 0.503 | 0.531 | 0.405 | 0.429 |  | 0.824 | 0.333 | 0.464 | 0.671 |
|  |  | (0.378) | (0.385) | (0.383) | (0.371) | (0.373) |  | (0.433)* | (0.380) | (0.388) | (0.388)* |
|  | Prefer not to say financial difficulties | 0.256 | 0.216 | 0.279 | 0.207 | 0.174 |  | 0.394 | 0.130 | 0.397 | 0.221 |
|  |  | (0.497) | (0.507) | (0.513) | (0.494) | (0.492) |  | (0.595) | (0.504) | (0.517) | (0.524) |
|  | EQ-5D score | -0.625 | -0.756 | -0.497 | -0.668 | -0.668 |  | -0.041 | -1.031 | -0.316 | -0.872 |
|  |  | (0.792) | (0.807) | (0.820) | (0.791) | (0.789) |  | (0.914) | (0.846) | (0.806) | (0.866) |
|  | Joint or not main carer | -1.175 | -1.448 | -1.482 | -1.452 | -1.305 |  | -0.833 | -1.429 | -1.358 | -1.191 |
|  |  | (0.697)* | (0.691)** | (0.690)** | (0.686)** | (0.690)* |  | (0.755) | (0.692)** | (0.710)* | (0.701)* |
|  | Total hours of care | 0.012 | 0.006 | 0.005 | 0.010 | 0.012 |  | 0.028 | 0.010 | 0.016 | 0.024 |
|  |  | (0.019) | (0.020) | (0.020) | (0.019) | (0.019) |  | (0.022) | (0.019) | (0.020) | (0.020) |
|  | Caring for 5 years or more | 0.096 | 0.077 | 0.090 | 0.175 | 0.137 |  | 0.226 | 0.232 | 0.254 | 0.058 |
|  |  | (0.303) | (0.313) | (0.313) | (0.300) | (0.300) |  | (0.349) | (0.311) | (0.319) | (0.314) |
|  | No replacement for a break | 0.383 | 0.451 | 0.404 | 0.436 | 0.430 |  | 0.675 | 0.448 | 0.447 | 0.406 |
|  |  | (0.281) | (0.291) | (0.291) | (0.279) | (0.281) |  | (0.327)** | (0.284) | (0.292) | (0.293) |
| Care recipient characteristics | 75 years old or older | -0.022 | -0.158 | -0.068 | -0.100 | -0.051 |  | 0.181 | -0.128 | 0.007 | 0.011 |
|  |  | (0.324) | (0.331) | (0.337) | (0.324) | (0.324) |  | (0.373) | (0.332) | (0.340) | (0.337) |
|  | Symptoms for 11 years or more | -0.254 | -0.204 | 0.028 | -0.242 | -0.237 |  | -0.021 | -0.384 | -0.307 | -0.184 |
|  |  | (0.520) | (0.546) | (0.543) | (0.518) | (0.518) |  | (0.560) | (0.526) | (0.538) | (0.533) |
|  | No diagnosis of dementia/don't know | -0.225 | -0.495 | -0.554 | -0.484 | -0.512 |  | -0.675 | -0.729 | -0.961 | -0.366 |
|  |  | (0.728) | (0.710) | (0.703) | (0.705) | (0.703) |  | (0.888) | (0.762) | (0.877) | (0.709) |
|  | Vascular dementia | 0.999 | 0.969 | 0.900 | 0.939 | 0.916 |  | 1.010 | 0.756 | 0.820 | 0.903 |
|  |  | (0.303)*** | (0.314)*** | (0.315)*** | (0.301)*** | (0.300)*** |  | (0.339)*** | (0.306)** | (0.310)*** | (0.311)*** |
|  | Other dementia | 0.113 | 0.062 | 0.116 | 0.101 | 0.096 |  | -0.086 | -0.035 | -0.005 | -0.114 |
|  |  | (0.325) | (0.328) | (0.331) | (0.323) | (0.325) |  | (0.370) | (0.333) | (0.337) | (0.339) |
|  | Moderate perceived severity | 0.425 | 0.440 | 0.363 | 0.411 | 0.398 |  | 0.113 | 0.392 | 0.321 | 0.249 |
|  |  | (0.466) | (0.470) | (0.474) | (0.467) | (0.471) |  | (0.517) | (0.471) | (0.487) | (0.474) |
|  | Severe perceived severity | 0.474 | 0.438 | 0.283 | 0.403 | 0.364 |  | -0.075 | 0.414 | 0.230 | 0.297 |
|  |  | (0.534) | (0.540) | (0.543) | (0.533) | (0.536) |  | (0.600) | (0.539) | (0.556) | (0.546) |
|  | Constant | -0.623 | -0.549 | -0.658 | -0.582 | -0.589 |  | -1.487 | -0.027 | -0.843 | -0.443 |
|  |  | (1.034) | (1.048) | (1.052) | (1.030) | (1.031) |  | (1.182) | (1.075) | (1.059) | (1.102) |
| Observations | | 282 | 274 | 268 | 287 | 285 |  | 227 | 269 | 259 | 266 |
| Most of the coefficients on dummies are interpreted in relation to the reference category. The reference categories for carer characteristics are respectively: Male, Younger than 75 years old, Below university education, No financial difficulties, Main carer status, Duration of caring under 5 years. The reference categories for the care recipient characteristics are: Younger than 74 years old, Duration of symptoms under 11 years, Mild severity of dementia. | | | | | | | | | | | |
| Robust standard errors in parentheses; ***=p-value<0.01, **=p-value<0.05, *=p-value<0.1 | | | | | | | | | | | |

Table S12 – First-stage results on the instrument of the IV approach.

| Instrumental Variable | ASCOT-Carer score | Self-efficacy on symptoms management | Self-efficacy on service use | Overall life satisfaction | Happiness yesterday | Overall costs | Carer's healthcare costs | Care recipient's healthcare costs | Care recipient's social care costs |
| --- | --- | --- | --- | --- | --- | --- | --- | --- | --- |
| Two-Stage Least Square estimator: first-stage estimated coefficient in main analysis | | | | | | | | | |
| Travel time | -1.314*** | -1.242*** | -1.303*** | -1.267*** | -1.284*** | -1.376*** | -1.354*** | -1.444*** | -1.277*** |
| CD F statistic | 48.153 | 41.741 | 41.138 | 45.057 | 45.341 | 47.155 | 49.597 | 56.661 | 43.216 |
| Two-Stage Least Square estimator: first-stage estimated coefficient in sensitivity analysis | | | | | | | | | |
| Travel time | -1.119*** | -1.088*** | -1.145*** | -1.127*** | -1.123*** |  |  |  |  |
| County LA | 0.418*** | 0.395*** | 0.428*** | 0.386*** | 0.399*** |  |  |  |  |
| London LA | 0.052 | -0.003 | -0.015 | -0.004 | 0.015 |  |  |  |  |
| Metropolitan LA | 0.090 | 0.086 | 0.080 | 0.068 | 0.071 |  |  |  |  |
| CD F statistic | 25.267 | 0.630 | 24.255 | 22.990 | 23.712 |  |  |  |  |
| SH test | 0.187 | 0.889 | 0.112 | 0.783 | 0.441 |  |  |  |  |
| LA=local authority; CD F statistic=Cragg-Donald F statistic; SH test=p-value of the Sargan-Hansen test; *** p<0.01, ** p<0.05, * p<0.1. | | | | | | | | | |

Table S13 – IV results when the logit regression uses fewer covariates.

| Variable | | ASCOT-Carer score | Self-efficacy on symptoms management | Self-efficacy on service use | Overall life satisfaction | Happiness yesterday |  | Overall costs | Carer's healthcare costs | Care recipient's healthcare costs | Social care costs |
| --- | --- | --- | --- | --- | --- | --- | --- | --- | --- | --- | --- |
|  | Admiral nursing | 1.206 | 1.692 | 3.048 | 0.126 | 1.022 |  | 78.250 | -37.698 | 132.775 | -339.518 |
|  |  | (0.832) | (2.980) | (2.519) | (0.644) | (0.641) |  | (430.475) | (182.731) | (226.054) | (317.961) |
| Carer characteristics | Female | -0.502 | -2.440 | -4.634 | -0.247 | -0.125 |  | -28.544 | 202.795 | 1.039 | -21.963 |
|  |  | (0.471) | (1.381)* | (1.223)*** | (0.304) | (0.311) |  | (200.534) | (174.873) | (139.468) | (164.331) |
|  | 75 years old or older | 0.330 | -0.670 | -3.701 | 0.657 | 0.540 |  | -25.490 | 221.863 | -14.220 | 35.980 |
|  |  | (0.504) | (1.541) | (1.216)*** | (0.334)** | (0.342) |  | (233.544) | (188.263) | (169.471) | (169.726) |
|  | Bachelor's degree | -0.536 | -2.250 | -2.561 | -0.434 | -0.462 |  | 395.374 | -125.189 | -107.790 | 376.839 |
|  |  | (0.451) | (1.353)* | (1.194)** | (0.282) | (0.299) |  | (205.187)* | (65.191)* | (92.935) | (148.976)** |
|  | Master's degree and above | -0.651 | -3.466 | -2.169 | -0.115 | 0.186 |  | 390.435 | -11.330 | -73.337 | 180.982 |
|  |  | (0.688) | (2.292) | (1.847) | (0.499) | (0.504) |  | (384.573) | (285.682) | (188.187) | (289.328) |
|  | Retired | 0.458 | 0.829 | 2.535 | 0.214 | 0.542 |  | 209.323 | 211.057 | 22.755 | 238.874 |
|  |  | (0.440) | (1.376) | (1.183)** | (0.284) | (0.304)* |  | (203.524) | (140.206) | (138.350) | (143.209)* |
|  | Some financial difficulties | -1.609 | -1.041 | -3.332 | -0.736 | -1.006 |  | 23.628 | -8.590 | 6.982 | -1.702 |
|  |  | (0.536)*** | (1.654) | (1.322)** | (0.341)** | (0.349)*** |  | (200.511) | (84.422) | (118.033) | (147.276) |
|  | Severe financial difficulties | -2.418 | -2.033 | -3.619 | -1.284 | -1.405 |  | 52.210 | 154.272 | -103.427 | 330.809 |
|  |  | (0.562)*** | (1.652) | (1.356)*** | (0.352)*** | (0.374)*** |  | (218.317) | (152.217) | (123.662) | (186.860)* |
|  | Prefer not to say financial difficulties | -2.903 | -1.502 | -4.510 | -0.795 | -1.218 |  | 731.766 | 393.986 | 398.984 | 240.235 |
|  |  | (0.753)*** | (2.176) | (1.789)** | (0.427)* | (0.468)*** |  | (433.377)* | (283.079) | (314.593) | (238.753) |
|  | EQ-5D score | 7.216 | 15.135 | 9.793 | 4.204 | 4.288 |  | 426.686 | -417.874 | -46.986 | 324.842 |
|  |  | (1.043)*** | (3.521)*** | (2.448)*** | (0.886)*** | (0.933)*** |  | (392.938) | (430.666) | (271.094) | (344.914) |
|  | Joint or not main carer | 0.854 | 4.433 | 3.272 | 1.060 | 0.941 |  | 295.323 | -142.682 | 173.247 | 110.006 |
|  |  | (0.868) | (1.748)** | (2.027) | (0.451)** | (0.467)** |  | (463.459) | (126.331) | (309.812) | (256.545) |
|  | Total hours of care | -0.105 | 0.247 | 0.092 | -0.022 | -0.027 |  | 10.382 | -11.737 | 12.825 | -8.790 |
|  |  | (0.027)*** | (0.082)*** | (0.073) | (0.016) | (0.017) |  | (13.143) | (5.933)** | (8.752) | (9.244) |
|  | Caring for 5 years or more | -0.468 | 0.150 | 0.112 | -0.248 | -0.083 |  | -172.137 | -165.672 | -412.900 | 313.522 |
|  |  | (0.422) | (1.360) | (1.046) | (0.258) | (0.296) |  | (217.238) | (102.202) | (156.512)*** | (161.671)* |
|  | No replacement for a break | -1.348 | -3.144 | -2.525 | -0.696 | -0.751 |  | 24.609 | -258.181 | -115.666 | 85.345 |
|  |  | (0.421)*** | (1.229)** | (1.031)** | (0.258)*** | (0.268)*** |  | (181.394) | (134.056)* | (127.202) | (136.017) |
| Care recipient characteristics | 75 years old or older | 0.405 | 2.800 | 2.688 | 0.081 | 0.094 |  | 160.927 | 43.467 | 203.690 | -47.528 |
|  |  | (0.443) | (1.484)* | (1.177)** | (0.296) | (0.322) |  | (191.720) | (78.937) | (132.626) | (140.259) |
|  | Symptoms for 11 years or more | 0.869 | 3.929 | 0.424 | 0.506 | 0.733 |  | 321.304 | -76.477 | 369.355 | -129.195 |
|  |  | (0.626) | (2.125)* | (1.840) | (0.382) | (0.541) |  | (399.610) | (242.323) | (318.194) | (264.733) |
|  | No diagnosis of dementia/don't know | -1.672 | -7.381 | -5.344 | -0.099 | -0.074 |  | 272.897 | -178.472 | 202.151 | -243.448 |
|  |  | (0.916)* | (2.229)*** | (2.472)** | (0.432) | (0.493) |  | (608.973) | (223.001) | (538.307) | (310.076) |
|  | Vascular dementia | -0.927 | -1.187 | -1.561 | -0.075 | 0.021 |  | -6.534 | 24.100 | 211.136 | -107.603 |
|  |  | (0.455)** | (1.405) | (1.181) | (0.296) | (0.310) |  | (199.681) | (118.761) | (118.339)* | (147.352) |
|  | Other dementia | -0.636 | -0.410 | -3.625 | -0.059 | 0.179 |  | 20.426 | 158.149 | 246.435 | -112.455 |
|  |  | (0.432) | (1.467) | (1.145)*** | (0.313) | (0.329) |  | (224.680) | (171.690) | (174.690) | (171.303) |
|  | Moderate perceived severity | -0.660 | -2.493 | -3.664 | -0.559 | -0.868 |  | -182.157 | 47.465 | -163.398 | 44.009 |
|  |  | (0.613) | (1.801) | (1.509)** | (0.392) | (0.415)** |  | (284.872) | (105.708) | (189.087) | (172.782) |
|  | Severe perceived severity | -2.037 | -4.358 | -4.017 | -1.261 | -1.543 |  | 546.178 | 477.390 | 94.181 | 561.977 |
|  |  | (0.689)*** | (2.158)** | (1.810)** | (0.472)*** | (0.497)*** |  | (357.364) | (253.730)* | (245.877) | (219.377)** |
|  | Constant | 8.933 | 17.963 | 22.821 | 3.335 | 3.257 |  | 65.113 | 332.547 | 118.415 | 43.584 |
|  |  | (1.428)*** | (4.407)*** | (3.333)*** | (1.099)*** | (1.170)*** |  | (558.894) | (539.411) | (408.036) | (424.088) |
| Observations | | 281 | 273 | 267 | 286 | 284 |  | 227 | 268 | 258 | 266 |
| R-squared | | 0.415 | 0.224 | 0.287 | 0.318 | 0.285 |  | 0.134 | 0.145 | 0.112 | 0.100 |
| Most of the coefficients on dummies are interpreted in relation to the reference category. The reference categories for carer characteristics are respectively: Male, Younger than 75 years old, Below university education, No financial difficulties, Main carer status, Duration of caring under 5 years. The reference categories for the care recipient characteristics are: Younger than 74 years old, Duration of symptoms under 11 years, Mild severity of dementia. | | | | | | | | | | | |
| Robust standard errors in parentheses; ***=p-value<0.01, **=p-value<0.05, *=p-value<0.1 | | | | | | | | | | | |

Figure S1 – Distribution of HRQoL and total hours of informal care.


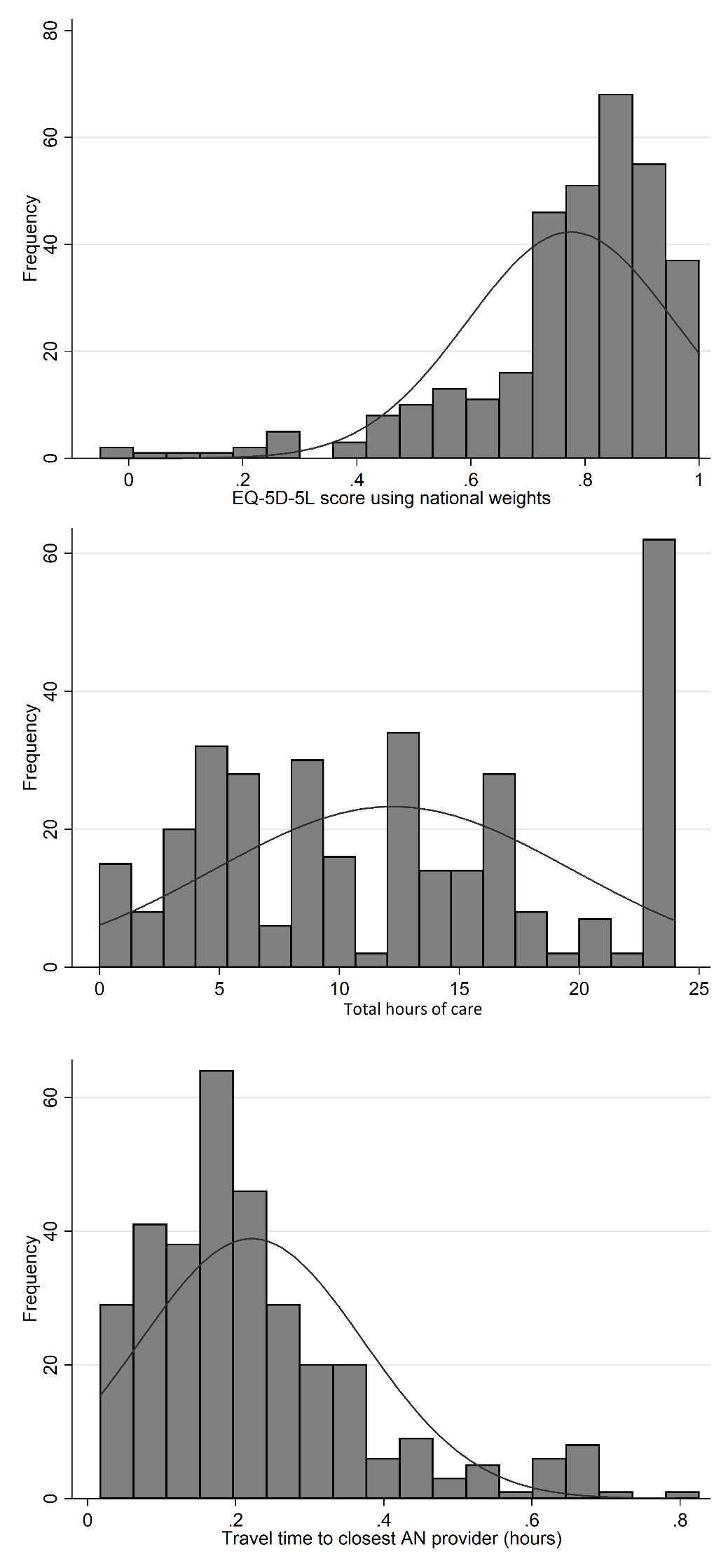


Figure S2 – Standardised difference in covariates before and after matching.


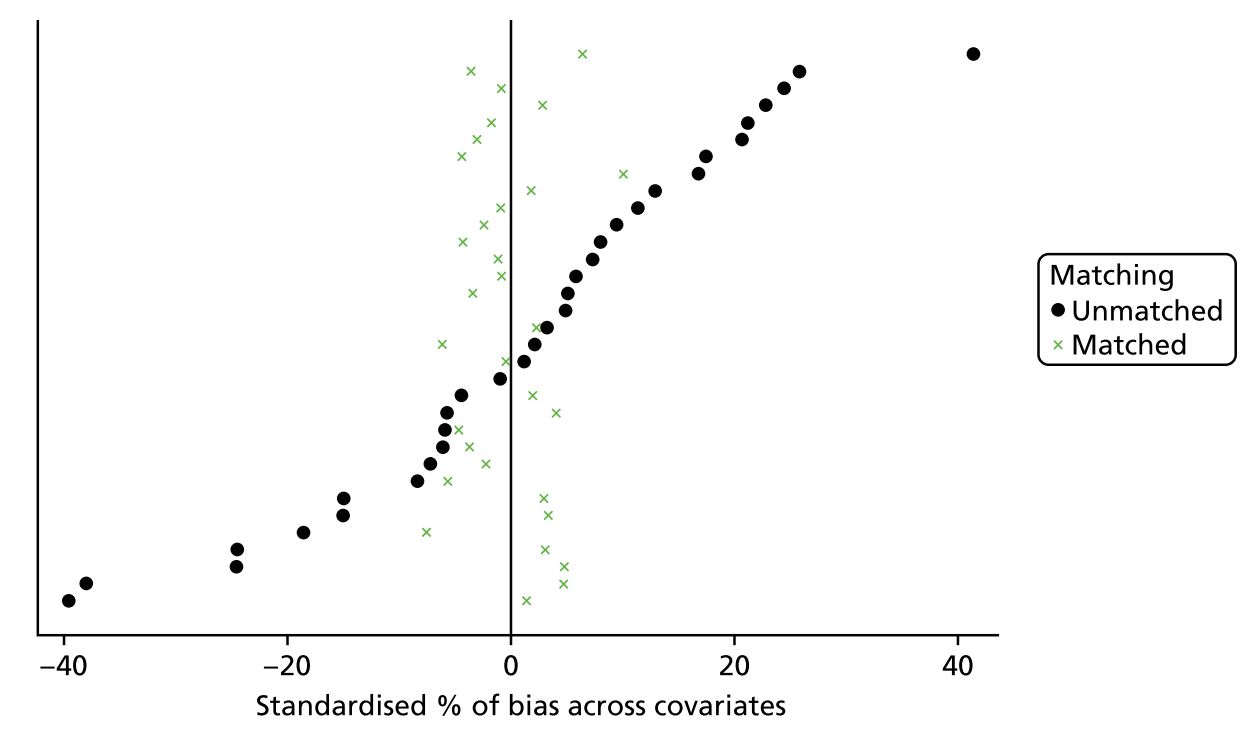


Figure S3 – Propensity score overlap before and after matching.


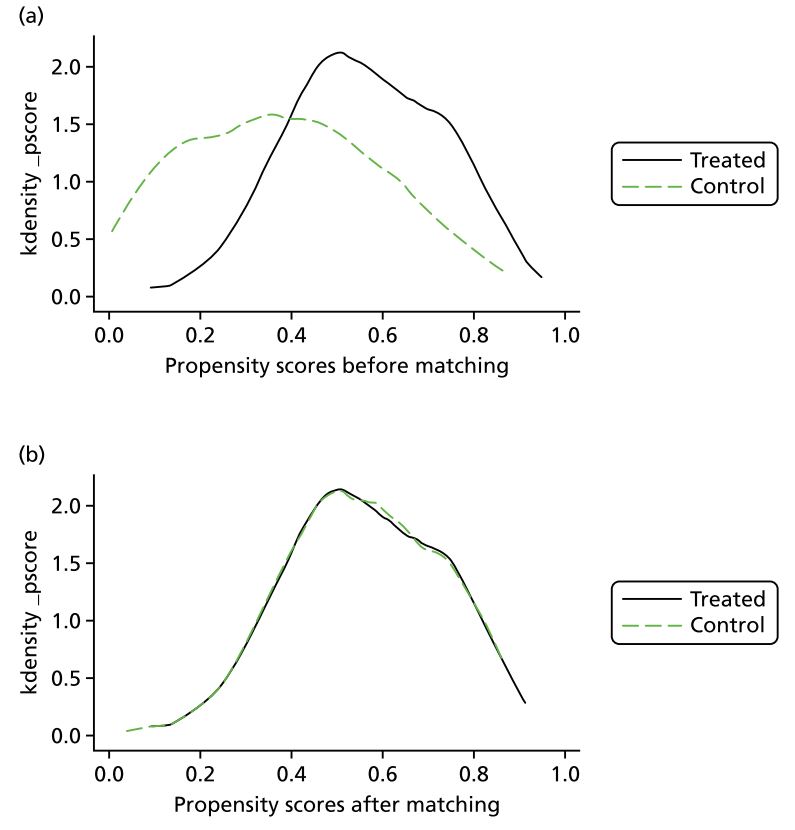


Figure S4 – Distribution of travel time.


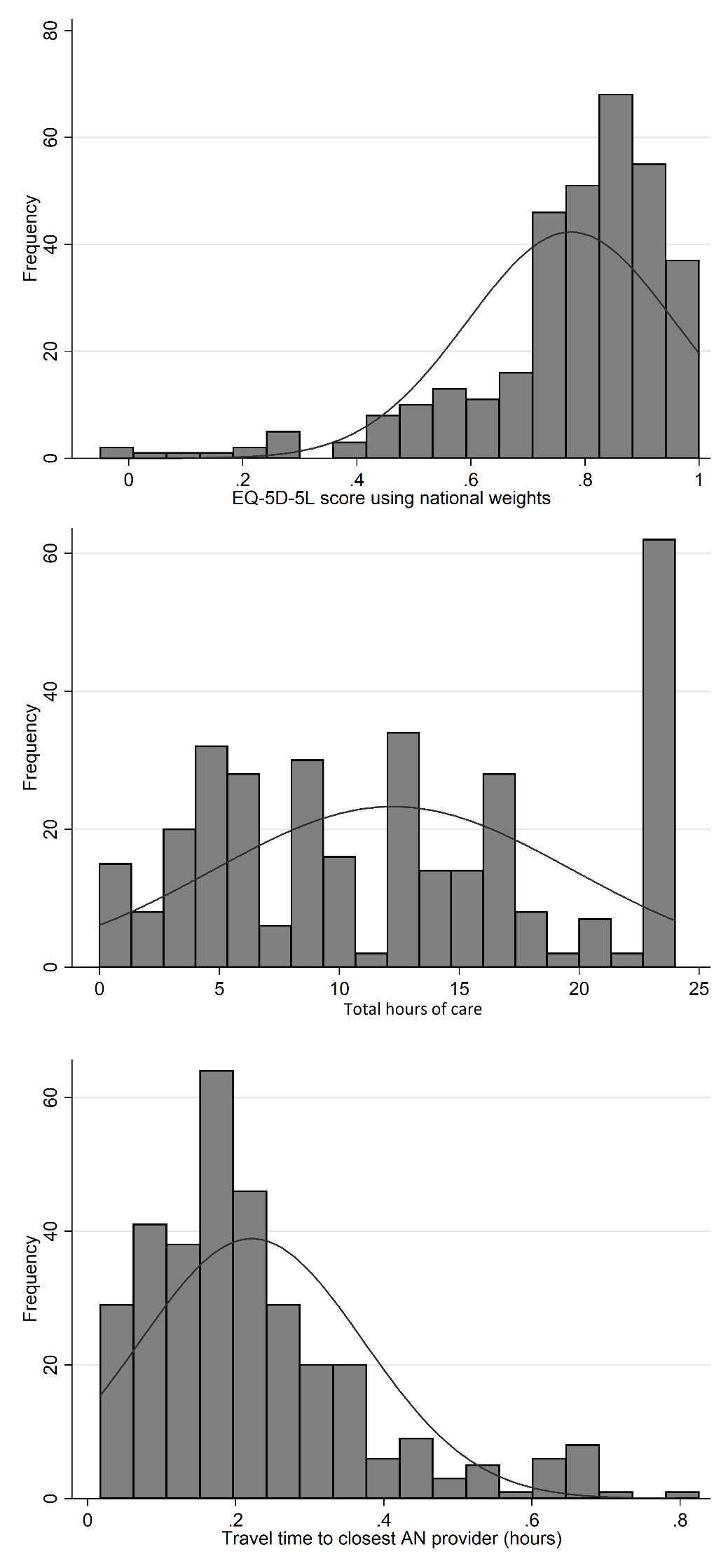

Supplement: Supplementary file 1 [file HSC-27-e734-s001.docx]
