# Supplementary material for: Investigating the economic case of a service to support carers of people with dementia: A cross‐sectional survey‐based feasibility study in England
Source: Health Soc Care Community. 2019 Jun 21;27(5):e734–43. doi: 10.1111/hsc.12799 (PMC6771746; doi:10.1111/hsc.12799)

Supporting Material

Table S1 – Unit costs of health and social care resources.

Item	Unit cost, £	Source	Notes
**Hospital services**			
Hospital outpatient appointment	120	NHS reference costs 2016	Average consultant led, non-consultant led and outpatient procedures.
Planned hospital admission without staying overnight	733	NHS reference costs 2016	Average day case
Planned hospital admission with an overnight stay	3750	NHS reference costs 2016	Average elective inpatient
Unplanned hospital admission	1609	NHS reference costs 2016	Trim point=<br>Average non-elective inpatient, including short stay
Other hospital admissions	389	NHS reference costs 2016	Regular day or night admissions
**Other health care services (rounded to units)**			
GP appointment	36	PSSRU 2016 p.154	Per surgery consultation of 9.22 minutes including direct care staff costs and qualification costs
Nurse appointment	11	PSSRU 2016 p.152<br><br>PSSRU 2015 p.183	Nurse based at GP practice including qualification costs: £44 per hour<br>Average contact duration is 15.5 minutes (PSSRU 2015)
Nurse specialist appointment	13	PSSRU 2016 p.151<br><br>PSSRU 2015 p.184	Nurse Band 7 including qualification costs: £52 per hour<br>Assumes Average contact duration is 15.5 minutes (PSSRU 2015) as per nurse based at GP practice.

Item	Unit cost, £	Source	Notes
Therapist appointment: occupational therapist, physiotherapist, speech therapist, chiropodist, podiatrist	9	PSSRU 2016 p.194	Allied health professionals Band 5: £34 per hour<br>Assumes Average contact duration is 15.5 minutes (PSSRU 2015) as per nurse based at GP practice.
**Social care services**			
Home care, per appointment	12	PSSRU 2016 p.169	Face-to-face weekday: £24 per hour<br>Assume 30 minutes per session (ref UKHCA 2016 report)
Day care, per hour	61	PSSRU 2016 p.37	£61 per client attendance
Meals, per meal	4	Glendenning *et al.* p.201; inflated to 2015-16	
Social service appointment: social worker	20	PSSRU 2016 p.165	Per hour of client-related work including qualifications=£79<br>Assumes Average contact duration is 15.5 minutes (PSSRU 2015) as per nurse based at GP practice.
Memory café, per session	14	Rotary club 2015	Based on example of memory café, at £138.33/monthly session. Assumes 10 carer-dementia pairs per session.

Table S2 – Unit costs used for costing informal care with proxy good method.

**Item**	**Unit cost, £**	**Source**	**Notes**	**Informal care activity**
Paid carer, per hour	24.6	PSSRU 2016 p.169	Weighted average of weekday, night and weekend hours.	Personal care, physical help, keeping company, transport, keeping an eye
Citizen Advice Bureau adviser, per hour	52	PSSRU 2016 p.171	Knapp *et al.*, 2013 used the cost of a family support worker to reflect the cost of an adviser.	Dealing with care services and benefits
Finance administrator, per hour	25	PSSRU 2016 p.146; NHS Agenda for change	Finance officer is at Agenda for change Band 3 (here).	Dealing with other paperwork and financial matters
Cleaner/handy person, per hour	21	PSSRU 2016 p.146	Housekeeping assistant is at Agenda for change Band 1. Mid-point Band 1 is £15,500/year. See here. Calculated from the ratio wages/hourly cost Band 2.	Practical help

Table S3 – Descriptive statistics on Admiral Nursing dummy and observed confounders.

	Covariate	All carers				AN		Non-AN	
		Freq	Mean	95% CI		Freq	Mean	Freq	Mean
Carer characteristics	Admiral nursing	132	0.468	0.410	0.527	132	1.000	0	0.000
	Male (ref)	87	0.309	0.257	0.365	45	0.341	42	0.280
	Female	195	0.691	0.635	0.743	87	0.659	108	0.720
	16 to 54 years old (ref)	50	0.177	0.137	0.227	16	0.121	34	0.227
	55 to 64 years old	71	0.252	0.204	0.306	29	0.220	42	0.280
	65 to 69 years old	31	0.110	0.078	0.152	17	0.129	14	0.093
	70 to 74 years old	42	0.149	0.112	0.196	22	0.167	20	0.133
	75 years or older	88	0.312	0.260	0.369	48	0.364	40	0.267
	Below university education (ref)	203	0.720	0.664	0.769	102	0.773	101	0.673
	Bachelor's degree	55	0.195	0.153	0.246	26	0.197	29	0.193
	Master's degree and above	24	0.085	0.058	0.124	4	0.030	20	0.133
	Full/part-time job	59	0.209	0.165	0.261	21	0.159	38	0.253
	Look after full time	70	0.248	0.201	0.302	39	0.295	31	0.207
	Retired	164	0.582	0.523	0.638	81	0.614	83	0.553
	No financial difficulties (ref)	66	0.234	0.188	0.287	26	0.197	40	0.267
	Some financial difficulties	92	0.326	0.274	0.383	41	0.311	51	0.340
	Severe financial difficulties	89	0.316	0.264	0.373	50	0.379	39	0.260
	Don't know/prefer not to say	35	0.124	0.090	0.168	15	0.114	20	0.133
	EQ-5D score	282	0.774	0.752	0.795	132	0.752	150	0.793
	Main carer (ref)	261	0.926	0.888	0.951	129	0.977	132	0.880
	Joint or not main carer	21	0.075	0.049	0.112	3	0.023	18	0.120
	Care recipient's spouse/partner (ref)	190	0.674	0.617	0.726	98	0.742	92	0.613
	Care recipient's child/child-in-law	81	0.287	0.237	0.343	30	0.227	51	0.340
	Other relationship with care recipient	11	0.039	0.022	0.069	4	0.030	7	0.047
	Personal care	198	0.702	0.646	0.753	99	0.750	99	0.660
	Physical care	136	0.482	0.424	0.541	71	0.538	65	0.433
	Total hours of care	282	12	11	13	132	13	150	11
	Caring for less than 1 year (ref)	18	0.064	0.040	0.099	6	0.046	12	0.080
	Caring for 1 to 3 years	91	0.323	0.270	0.380	48	0.364	43	0.287
	Caring for 3 to 5 years	78	0.277	0.227	0.332	31	0.235	47	0.313
	Caring for 5 to 10 years	66	0.234	0.188	0.287	33	0.250	33	0.220
	Caring for 10 years or more	29	0.103	0.072	0.144	14	0.106	15	0.100
	Replacement for a break (ref)	116	0.411	0.355	0.470	43	0.326	73	0.487
	No replacement for a break	166	0.589	0.530	0.645	89	0.674	77	0.513
Care recipient characteristics	45 to 64 years old (ref)	28	0.099	0.069	0.140	10	0.076	18	0.120
	65 to 74 years old	57	0.202	0.159	0.253	28	0.212	29	0.193
	75 years or older old	197	0.699	0.642	0.750	94	0.712	103	0.687
	Duration of symptoms under 1 year (ref)	13	0.046	0.027	0.078	8	0.061	5	0.033
	Duration of symptoms from 1 to 5 years	166	0.589	0.530	0.645	79	0.598	87	0.580
	Duration of symptoms from 6 to 10 years	79	0.280	0.231	0.336	35	0.265	44	0.293
	Duration of symptoms for 11 years or more	24	0.085	0.058	0.124	10	0.076	14	0.093
	Formal diagnosis of dementia (ref)	272	0.965	0.935	0.981	128	0.970	144	0.960
	No diagnosis of dementia/don't know	10	0.036	0.019	0.065	4	0.030	6	0.040
	Alzheimer's	171	0.606	0.548	0.662	68	0.515	103	0.687
	Vascular dementia	94	0.333	0.280	0.391	58	0.439	36	0.240
	Other dementia	76	0.270	0.221	0.325	34	0.258	42	0.280
	Mild perceived severity (ref)	29	0.103	0.072	0.144	9	0.068	20	0.133
	Moderate perceived severity	178	0.631	0.573	0.686	85	0.644	93	0.620
	Severe perceived severity	75	0.266	0.217	0.321	38	0.288	37	0.247

Obs=total number of observations; Freq=number of observations for each category (it is equivalent to Obs for continuous variables); Mean=proportion for dummy variables and average for continuous variables; CI=confidence intervals; ref=reference category.

The sample included 282 carers, of which 132 AN carers and 150 non-AN carers. The descriptive statistics reported in this table were calculated on the ASCOT-Carer score's sample.

Table S4 – Full results of the regression analysis on outcomes.

	Variable	ASCOT-Carer score	Self-efficacy on symptoms management	Self-efficacy on service use	Overall life satisfaction	Happiness yesterday
	Admiral nursing	0.382<br>(0.397)	1.243<br>(1.317)	1.990*<br>(1.060)	0.087<br>(0.266)	0.433<br>(0.279)
Carer characteristics	Female	-0.539<br>(0.495)	-2.384<br>(1.446)	-3.842***<br>(1.232)	-0.166<br>(0.334)	0.001<br>(0.344)
	55 to 64 years old	-0.134<br>(0.705)	-2.399<br>(1.982)	-1.712<br>(1.853)	-0.387<br>(0.439)	-0.618<br>(0.466)
	65 to 69 years old	0.127<br>(0.979)	-2.214<br>(3.030)	0.596<br>(2.805)	-0.181<br>(0.664)	-0.300<br>(0.719)
	70 to 74 years old	0.202<br>(0.903)	-1.761<br>(2.777)	1.448<br>(2.510)	-0.015<br>(0.641)	0.227<br>(0.654)
	75 years old or older	0.833<br>(1.023)	-3.149<br>(3.090)	-5.370*<br>(2.796)	0.629<br>(0.721)	0.545<br>(0.735)
	Bachelor's degree	-0.483<br>(0.444)	-2.408*<br>(1.441)	-2.403*<br>(1.293)	-0.456<br>(0.300)	-0.467<br>(0.313)
	Master's degree and above	-0.871<br>(0.685)	-3.096<br>(2.494)	-1.205<br>(1.849)	-0.083<br>(0.508)	0.157<br>(0.540)
	Full/part-time job	1.212*<br>(0.663)	2.045<br>(2.039)	1.227<br>(1.886)	0.069<br>(0.401)	-0.042<br>(0.430)
	Look after full time	0.273<br>(0.486)	0.264<br>(1.534)	0.743<br>(1.397)	0.050<br>(0.319)	0.055<br>(0.346)
	Retired	1.159**<br>(0.564)	2.216<br>(1.893)	2.152<br>(1.643)	0.289<br>(0.376)	0.499<br>(0.403)
	Some financial difficulties	-1.531***<br>(0.580)	-1.384<br>(1.802)	-3.928***<br>(1.419)	-0.830**<br>(0.378)	-1.075***<br>(0.389)
	Severe financial difficulties	-2.281***<br>(0.605)	-2.936<br>(1.800)	-3.926***<br>(1.399)	-1.308***<br>(0.393)	-1.382***<br>(0.403)
	Don't know/prefer not to say	-2.636***<br>(0.782)	-1.904<br>(2.338)	-4.371**<br>(1.837)	-0.740<br>(0.472)	-1.136**<br>(0.490)
	EQ-5D score	7.131***<br>(1.145)	13.295***<br>(3.722)	8.581***<br>(2.887)	4.129***<br>(0.934)	4.160***<br>(0.957)
	Joint or not main carer	0.543<br>(0.962)	2.790<br>(1.898)	3.307<br>(2.112)	0.823*<br>(0.483)	0.535<br>(0.484)
	Care recipient's child/child-in-law	0.889<br>(0.818)	0.375<br>(2.534)	-2.327<br>(2.211)	0.336<br>(0.570)	0.407<br>(0.603)
	Other relationship with care recipient	0.063<br>(1.262)	-4.703<br>(3.610)	-5.352*<br>(3.094)	-0.200<br>(0.806)	-0.206<br>(0.720)
	Personal care	-0.495<br>(0.505)	-1.076<br>(1.553)	-0.896<br>(1.294)	-0.214<br>(0.314)	-0.031<br>(0.350)
	Physical care	-0.578<br>(0.468)	2.578*<br>(1.436)	0.964<br>(1.201)	0.108<br>(0.319)	0.003<br>(0.344)
	Total hours of care	-0.084***<br>(0.030)	0.213**<br>(0.094)	0.042<br>(0.081)	-0.023<br>(0.018)	-0.029<br>(0.019)
	Caring for 1 to 3 years	-0.142<br>(1.082)	2.512<br>(2.976)	6.458***<br>(2.160)	0.274<br>(0.719)	0.765<br>(0.669)
	Caring for 3 to 5 years	-0.195<br>(1.142)	1.496<br>(3.174)	4.667**<br>(2.213)	0.331<br>(0.742)	0.895<br>(0.710)
	Caring for 5 to 10 years	-0.108<br>(1.211)	2.149<br>(3.386)	5.895**<br>(2.397)	-0.141<br>(0.771)	0.453<br>(0.774)
	Caring for 10 years or more	-0.658<br>(1.342)	0.493<br>(4.155)	4.568<br>(3.045)	-0.021<br>(0.845)	0.360<br>(0.892)
	No replacement for a break	-1.192***<br>(0.440)	-3.433**<br>(1.341)	-2.829***<br>(1.083)	-0.746***<br>(0.281)	-0.772***<br>(0.293)
Care recipient characteristics	65 to 74 years old	0.782<br>(0.708)	0.187<br>(2.736)	1.552<br>(2.078)	0.529<br>(0.507)	0.640<br>(0.520)
	75 years old or older	0.665<br>(0.762)	2.396<br>(2.884)	4.932**<br>(2.095)	0.290<br>(0.575)	0.367<br>(0.563)
	Symptoms for 1 to 5 years	0.107<br>(1.175)	1.565<br>(3.644)	0.402<br>(3.661)	0.961<br>(0.760)	-0.185<br>(0.635)
	Symptoms for 6 to 10 years	-0.247<br>(1.289)	2.725<br>(4.091)	0.117<br>(4.004)	1.260<br>(0.861)	0.429<br>(0.798)

Variable	ASCOT-Carer score	Self-efficacy on symptoms management	Self-efficacy on service use	Overall life satisfaction	Happiness yesterday
Symptoms for 11 years or more	0.993	6.705	1.174	1.567*	0.985
	(1.450)	(4.809)	(4.508)	(0.910)	(0.953)
No diagnosis of dementia/don't know	-1.424	-6.734***	-4.891*	-0.208	-0.329
	(0.926)	(2.574)	(2.584)	(0.454)	(0.486)
Vascular dementia	-0.722	-1.211	-1.682	-0.096	0.159
	(0.445)	(1.373)	(1.148)	(0.308)	(0.326)
Other dementia	-0.561	-0.827	-4.162***	-0.062	0.218
	(0.450)	(1.618)	(1.308)	(0.322)	(0.338)
Moderate perceived severity	-0.585	-2.828	-3.692**	-0.827*	-1.012*
	(0.715)	(2.080)	(1.688)	(0.474)	(0.531)
Severe perceived severity	-1.722**	-5.450**	-4.208**	-1.574***	-1.795***
	(0.783)	(2.533)	(2.066)	(0.552)	(0.597)
Constant	7.928***	17.986***	18.970***	2.271	2.782*
	(2.007)	(5.858)	(4.716)	(1.435)	(1.482)
Observations	282	274	268	287	285
R-squared	0.459	0.260	0.350	0.340	0.335

Most of the coefficients on dummies are interpreted in relation to the reference category. The reference categories for carer characteristics are respectively: Male, Age 16 to 54 years, Below university education, No financial difficulties. The reference categories for the characteristics of the caring role are: Main carer status, Person with dementia is spouse/partner, Duration of caring under 1 year. Finally, the reference categories for the care recipient characteristics are: Age 45 to 64, Duration of symptoms under 1 year, Severity of dementia is Mild. Table 3 shows the reference categories.

Robust standard errors in parentheses; ***=p-value<0.01, **=p-value<0.05, *=p-value<0.1

Table S5 – Regression and IV results after clustering standard errors or using local authority random effects.

	ASCOT-Carer score	Self-efficacy on symptoms management	Self-efficacy on service use	Overall life satisfaction	Happiness yesterday	Overall costs	Carer's healthcare costs	Care recipient's healthcare costs	Social care costs
	Regression with clustered standard errors within local authorities								
Coeff	0.361	1.020	1.651	0.047	0.375	46.455	-183.693	-105.097	77.329
Std Err	(0.357)	(1.454)	(1.286)	(0.318)	(0.363)	(193.965)	(182.582)	(139.727)	(105.901)
	Regression with local authority random effects and clustered standard errors								
Coeff	0.361	1.020	1.078	0.103	0.490	46.455	-178.775	-48.940	77.333
Std Err	(0.333)	(1.353)	(1.370)	(0.343)	(0.397)	(177.772)	(151.431)	(225.510)	(98.388)
	Instrumental Variables with clustered standard errors within local authorities								
Coeff	1.472	2.899	2.586	0.174	0.937	-29.413	-44.331	187.689	-440.286
Std Err	(0.874)*	(2.651)	(2.772)	(0.461)	(0.454)**	(336.732)	(265.401)	(209.150)	(293.622)

Coeff=estimated coefficient on the Admiral Nursing dummy; Std Err=robust standard errors.
In all regressions, we controlled for carer characteristics (including gender, age, education, work situation, household financial difficulties, whether the carer was a sole carer, relationship with the care recipient, type and amount of time of care provided, number of years caring, availability of a replacement for a break, and HRQoL) and care recipient characteristics (including age, duration of symptoms of dementia, existence of a formal diagnosis, type of dementia such as Alzheimer, vascular dementia, or other type of dementia, and perceived severity of dementia). In the IV regression, the instrument was the travel time to the closest AN provider.
Clustered standard errors in parenthesis; ***=p-value<0.01, **=p-value<0.05, *=p-value<0.1

Table S6 – Regression results using GLM for outcomes and two-part model for costs.

	ASCOT-Carer score	Self-efficacy on symptoms management	Self-efficacy on service use	Overall life satisfaction	Happiness yesterday	Overall costs	Carer's healthcare costs	Care recipient's healthcare costs	Social care costs
	GLM regression with log link and normal distribution					Two-part model (logit, GLM with log link and normal distribution)			
Coeff	0.168	0.836	1.569	0.078	0.295	192.415	-	-	266.575
Std Err	(0.404)	(1.214)	(0.987)	(0.249)	(0.251)	(582.366)	-	-	(323.136)
	GLM regression with log link and gamma distribution					Two-part model (logit, GLM with log link and gamma distribution)			
Coeff	0.689	1.663	2.522	0.062	0.613	409.218	-55.264	-54.377	118.241
Std Err	(0.425)	(1.401)	(1.175)**	(0.314)	(0.339)*	(252.277)	(79.839)	(93.319)	(151.750)

Coeff=estimated coefficient on the Admiral Nursing dummy; Std Err=robust standard errors.

The two-part model using GLM with log link and normal distribution in the second part did not converge when the dependent variable was the carer's healthcare costs and the care recipient's healthcare costs. Estimates are therefore omitted.

In all regressions, we controlled for carer characteristics (including gender, age, education, work situation, household financial difficulties, whether the carer was a sole carer, relationship with the care recipient, type and amount of time of care provided, number of years caring, availability of a replacement for a break, and HRQoL) and care recipient characteristics (including age, duration of symptoms of dementia, existence of a formal diagnosis, type of dementia such as Alzheimer, vascular dementia, or other type of dementia, and perceived severity of dementia). In the IV regression, the instrument was the travel time to the closest AN provider.

Robust standard errors in parenthesis; ***=p-value<0.01, **=p-value<0.05, *=p-value<0.1

Table S7 – Missing data.

Variable	Resp	Miss	Prop
Outcome			
ASCOT-Carer score	317	29	8.4%
Self-efficacy on symptoms management	310	36	10.4%
Self-efficacy on service use	302	44	12.7%
Overall life satisfaction	330	16	4.6%
Happiness yesterday	328	18	5.2%
Costs			
Overall costs	260	86	24.9%
Carer's healthcare costs	306	40	11.6%
Care recipient's healthcare costs	297	49	14.2%
Social care costs	307	39	11.3%
Carer characteristics			
Gender	340	6	1.7%
Age	340	6	1.7%
Education	339	7	2.0%
Work situation	346	0	0.0%
Household financial difficulties	316	30	8.7%
EQ-5D score	330	16	4.6%
Caring status	336	10	2.9%
Relationship with the care recipient	333	13	3.8%
Provision of personal care	346	0	0.0%
Provision of physical care	346	0	0.0%
Total hours of caring	328	18	5.2%
Duration of caring	339	7	2.0%
Availability of a replacement for a break	337	9	2.6%
Care recipient characteristics			
Age	331	15	4.3%
Duration of symptoms of dementia	339	7	2.0%
Formal diagnosis of dementia	340	6	1.7%
Type of dementia	346	0	0.0%
Perceived severity of dementia	339	7	2.0%
Instruments			
Travel time	327	19	5.5%
Unitary LA	327	19	5.5%
County LA	327	19	5.5%
London LA	327	19	5.5%
Metropolitan LA	327	19	5.5%

Resp=number of respondents, Miss=Number of missing responses, Prop=proportion of missing responses.
Numbers and proportions are calculated considering the 346 in-scope questionnaires.

Table S8 – Regression results using fewer covariates.

	Variable	ASCOT-Carer score	Self-efficacy on symptoms management	Self-efficacy on service use	Overall life satisfaction	Happiness yesterday	Overall costs	Carer's healthcare costs	Care recipient's healthcare costs	Social care costs
	Admiral nursing	0.290	0.986	2.334**	0.008	0.414	59.667	-149.215	-90.373	62.143
		(0.381)	(1.273)	(1.033)	(0.258)	(0.270)	(194.057)	(125.449)	(129.852)	(143.216)
Carer characteristics	Female	-0.565	-2.293	-4.517***	-0.242	-0.128	-30.147	202.421	-1.196	9.332
		(0.478)	(1.449)	(1.248)	(0.316)	(0.320)	(207.972)	(183.805)	(145.176)	(169.158)
	75 years old or older	0.366	-0.739	-3.738***	0.658*	0.562	-25.753	219.301	-4.414	29.910
		(0.524)	(1.574)	(1.281)	(0.341)	(0.347)	(245.002)	(192.749)	(174.937)	(172.547)
	Bachelor's degree	-0.543	-2.193	-2.523**	-0.431	-0.460	395.924*	-125.268*	-101.558	377.812**
		(0.459)	(1.399)	(1.244)	(0.292)	(0.305)	(217.122)	(68.130)	(99.827)	(156.532)
	Master's degree and above	-0.925	-3.516	-2.270	-0.138	0.038	383.993	-35.129	-129.931	314.823
		(0.661)	(2.356)	(1.869)	(0.481)	(0.510)	(376.738)	(270.650)	(187.357)	(257.183)
	Retired	0.453	0.768	2.513**	0.211	0.530*	209.308	211.766	19.017	242.305*
		(0.447)	(1.427)	(1.237)	(0.293)	(0.309)	(214.228)	(145.108)	(141.940)	(142.832)
	Some financial difficulties	-1.595***	-1.059	-3.342**	-0.737**	-1.006***	25.096	-10.190	9.388	-27.000
		(0.551)	(1.719)	(1.372)	(0.355)	(0.360)	(207.259)	(89.092)	(120.851)	(149.520)
	Severe financial difficulties	-2.319***	-2.207	-3.713***	-1.287***	-1.379***	55.588	155.485	-87.986	270.256
		(0.563)	(1.709)	(1.378)	(0.365)	(0.376)	(204.679)	(154.623)	(119.780)	(174.600)
	Prefer not to say financial difficulties	-2.857***	-1.498	-4.474**	-0.792*	-1.200**	733.524	396.569	416.838	219.481
		(0.773)	(2.254)	(1.838)	(0.444)	(0.481)	(458.802)	(293.583)	(324.569)	(240.939)
	EQ-5D score	7.096***	14.795***	9.571***	4.174***	4.169***	426.484	-448.593	-69.056	398.446
		(1.076)	(3.652)	(2.527)	(0.918)	(0.946)	(413.041)	(458.009)	(266.576)	(336.976)
	Joint or not main carer	0.694	4.135**	3.000	1.026**	0.797*	292.988	-172.807	120.858	186.536
		(0.904)	(1.708)	(2.074)	(0.452)	(0.478)	(481.382)	(139.352)	(320.606)	(252.849)
	Total hours of care	-0.102***	0.235***	0.082	-0.023	-0.027	10.482	-11.903*	13.182	-10.776
		(0.028)	(0.087)	(0.077)	(0.017)	(0.017)	(13.672)	(6.296)	(9.195)	(9.575)
	Caring for 5 years or more	-0.459	0.355	0.257	-0.234	-0.043	-171.311	-155.215	-395.307**	309.536*
		(0.432)	(1.429)	(1.094)	(0.269)	(0.304)	(223.739)	(104.922)	(154.709)	(167.524)
	No replacement for a break	-1.275***	-3.091**	-2.469**	-0.686**	-0.700**	27.120	-248.147*	-95.026	52.929
		(0.427)	(1.262)	(1.055)	(0.267)	(0.276)	(191.521)	(135.473)	(134.575)	(143.035)
Care recipient characteristics	75 years old or older	0.418	2.560*	2.516**	0.067	0.065	161.804	34.627	198.617	-53.246
		(0.458)	(1.540)	(1.229)	(0.306)	(0.331)	(204.810)	(80.891)	(137.661)	(143.388)
	Symptoms for 11 years or more	0.811	4.123*	0.574	0.511	0.726	321.109	-80.269	360.467	-111.081
		(0.632)	(2.227)	(1.912)	(0.405)	(0.553)	(419.557)	(244.166)	(321.130)	(271.786)

Variable	ASCOT-Carer score	Self-efficacy on symptoms management	Self-efficacy on service use	Overall life satisfaction	Happiness yesterday	Overall costs	Carer's healthcare costs	Care recipient's healthcare costs	Social care costs
No diagnosis of dementia/don't know	-1.729*	-7.323***	-5.335**	-0.103	-0.124	270.059	-190.345	162.840	-209.160
	(0.953)	(2.323)	(2.601)	(0.441)	(0.510)	(647.331)	(230.279)	(588.173)	(312.728)
Vascular dementia	-0.733*	-0.972	-1.369	-0.048	0.148	-2.642	43.662	251.909**	-183.553
	(0.425)	(1.320)	(1.158)	(0.288)	(0.299)	(188.934)	(113.589)	(116.078)	(144.196)
Other dementia	-0.606	-0.686	-3.824***	-0.075	0.148	20.163	147.075	235.617	-102.371
	(0.442)	(1.545)	(1.211)	(0.321)	(0.336)	(235.290)	(175.882)	(177.272)	(175.228)
Moderate perceived severity	-0.570	-2.420	-3.591**	-0.548	-0.813*	-181.598	57.881	-147.063	19.629
	(0.641)	(1.838)	(1.539)	(0.401)	(0.431)	(301.172)	(111.574)	(197.803)	(157.864)
Severe perceived severity	-1.930***	-4.637**	-4.218**	-1.271***	-1.541***	546.147	477.513*	95.133	532.823**
	(0.718)	(2.231)	(1.880)	(0.482)	(0.515)	(376.130)	(259.581)	(254.283)	(211.172)
Constant	9.246***	18.735***	23.400***	3.408***	3.548***	68.503	401.477	204.128	-115.178
	(1.473)	(4.486)	(3.315)	(1.136)	(1.188)	(570.341)	(569.224)	(394.070)	(394.955)
Observations	282	274	268	287	285	227	269	259	266
R-squared	0.428	0.222	0.289	0.320	0.303	0.134	0.147	0.123	0.134

Most of the coefficients on dummies are interpreted in relation to the reference category. The reference categories for carer characteristics are respectively: Male, Younger than 75 years old, Below university education, No financial difficulties, Main carer status, Duration of caring under 5 years. The reference categories for the care recipient characteristics are: Younger than 74 years old, Duration of symptoms under 11 years, Mild severity of dementia.

Robust standard errors in parentheses; ***=p-value<0.01, **=p-value<0.05, *=p-value<0.1

Table S9 – Logit regression on the Admiral Nursing dummy.

	Variable	Admiral Nursing
Carer's characteristics	Female	0.816
	55 to 64 years old	1.905
	65 to 69 years old	3.129
	70 to 74 years old	2.036
	75 years old or older	1.935
	Bachelor's degree	1.015
	Master's degree and above	0.222**
	Full/part-time job	1.115
	Look after full time	1.241
	Retired	0.832
	Some financial difficulties	1.088
	Severe financial difficulties	1.697
	Don't know/prefer not to say	1.258
	EQ-5D score	0.483
Caring role	Joint or not main carer	0.398
	Child/child-in-law	0.829
	Other relationship	1.154
	Personal care	1.300
	Physical care	1.117
	Total hours of care	1.009
	Caring for 1 to 3 years	3.834*
	Caring for 3 to 5 years	2.327
	Caring for 5 to 10 years	3.860
	Caring for 10 years or more	3.871
	No replacement for a break	1.512
Care recipient's characteristics	65 to 74 years old	0.986
	75 years old or older	1.132
	Symptoms for 1 to 5 years	0.135**
	Symptoms for 6 to 10 years	0.084**
	Symptoms for 11 years or more	0.067**
	No diagnosis/don't know	0.668
	Vascular dementia	2.456***
	Other dementia	1.055
	Moderate severity	2.367
	Severe severity	2.444
	Constant	0.440
Observations		282

Logit regression analysis on ASCOT-Carer score's sample. The Admiral Nursing dummy is the dependent variable. Most of the coefficients on dummies are interpreted in relation to the reference category. Table 3 shows the reference categories.

Robust standard errors in parentheses; *** p<0.01, ** p<0.05, * p<0.1.

Table S10 – Comparison between PSM techniques on average standardised difference of the covariates.

Technique	Sample	Mean bias	Median bias
Kernel	Unmatched	14.4	11.4
	Matched	3.4	3.1
Nearest neighbour	Unmatched	14.4	11.4
	Matched	8.4	7.6
Calliper (0.2)	Unmatched	14.4	11.4
	Matched	8.4	7.6

Table S11 – PSM results when the logit regression uses fewer covariates.

	Variable	ASCOT-Carer score	Self-efficacy on symptoms management	Self-efficacy on service use	Overall life satisfaction	Happiness yesterday	Overall costs	Carer's healthcare costs	Care recipient's healthcare costs	Social care costs
	Admiral nursing	0.538	1.474	2.937	0.133	0.541	-43.233	-175.572	-130.078	-12.104
		(0.527)	(1.471)	(1.288)**	(0.314)	(0.330)	(211.553)	(129.353)	(132.273)	(137.414)
	Estimated coefficients from Logit regression in which the AN dummy is the dependent variable									
Carer characteristics	Female	-0.296	-0.185	-0.234	-0.236	-0.211	-0.419	-0.256	-0.168	-0.367
		(0.322)	(0.327)	(0.326)	(0.317)	(0.318)	(0.361)	(0.327)	(0.327)	(0.338)
	75 years old or older	0.203	0.376	0.287	0.337	0.322	-0.059	0.088	0.288	0.104
		(0.355)	(0.366)	(0.366)	(0.355)	(0.356)	(0.415)	(0.364)	(0.374)	(0.369)
	Bachelor's degree	-0.030	0.042	-0.028	0.002	-0.023	0.119	-0.045	0.095	-0.009
		(0.334)	(0.338)	(0.339)	(0.333)	(0.333)	(0.386)	(0.342)	(0.361)	(0.340)
	Master's degree and above	-1.660	-1.944	-1.953	-1.603	-1.623	-2.150	-1.481	-1.515	-1.933
		(0.621)***	(0.690)***	(0.685)***	(0.620)***	(0.618)***	(0.848)**	(0.633)**	(0.629)**	(0.708)***
	Retired	-0.047	-0.016	0.040	-0.063	-0.062	-0.001	0.068	-0.059	-0.049
		(0.315)	(0.324)	(0.327)	(0.316)	(0.316)	(0.379)	(0.320)	(0.333)	(0.332)
	Some financial difficulties	0.104	0.154	0.151	0.086	0.079	0.392	0.015	0.111	0.325
		(0.364)	(0.373)	(0.372)	(0.360)	(0.361)	(0.413)	(0.368)	(0.374)	(0.379)
	Severe financial difficulties	0.446	0.503	0.531	0.405	0.429	0.824	0.333	0.464	0.671
		(0.378)	(0.385)	(0.383)	(0.371)	(0.373)	(0.433)*	(0.380)	(0.388)	(0.388)*
	Prefer not to say financial difficulties	0.256	0.216	0.279	0.207	0.174	0.394	0.130	0.397	0.221
		(0.497)	(0.507)	(0.513)	(0.494)	(0.492)	(0.595)	(0.504)	(0.517)	(0.524)
	EQ-5D score	-0.625	-0.756	-0.497	-0.668	-0.668	-0.041	-1.031	-0.316	-0.872
		(0.792)	(0.807)	(0.820)	(0.791)	(0.789)	(0.914)	(0.846)	(0.806)	(0.866)
	Joint or not main carer	-1.175	-1.448	-1.482	-1.452	-1.305	-0.833	-1.429	-1.358	-1.191
		(0.697)*	(0.691)**	(0.690)**	(0.686)**	(0.690)*	(0.755)	(0.692)**	(0.710)*	(0.701)*
	Total hours of care	0.012	0.006	0.005	0.010	0.012	0.028	0.010	0.016	0.024
		(0.019)	(0.020)	(0.020)	(0.019)	(0.019)	(0.022)	(0.019)	(0.020)	(0.020)
	Caring for 5 years or more	0.096	0.077	0.090	0.175	0.137	0.226	0.232	0.254	0.058
		(0.303)	(0.313)	(0.313)	(0.300)	(0.300)	(0.349)	(0.311)	(0.319)	(0.314)
	No replacement for a break	0.383	0.451	0.404	0.436	0.430	0.675	0.448	0.447	0.406
		(0.281)	(0.291)	(0.291)	(0.279)	(0.281)	(0.327)**	(0.284)	(0.292)	(0.293)
Care recipie[illegible]	75 years old or older	-0.022	-0.158	-0.068	-0.100	-0.051	0.181	-0.128	0.007	0.011
		(0.324)	(0.331)	(0.337)	(0.324)	(0.324)	(0.373)	(0.332)	(0.340)	(0.337)

Variable	ASCOT-Carer score	Self-efficacy on symptoms management	Self-efficacy on service use	Overall life satisfaction	Happiness yesterday	Overall costs	Carer's healthcare costs	Care recipient's healthcare costs	Social care costs
Symptoms for 11 years or more	-0.254	-0.204	0.028	-0.242	-0.237	-0.021	-0.384	-0.307	-0.184
	(0.520)	(0.546)	(0.543)	(0.518)	(0.518)	(0.560)	(0.526)	(0.538)	(0.533)
No diagnosis of dementia/don't know	-0.225	-0.495	-0.554	-0.484	-0.512	-0.675	-0.729	-0.961	-0.366
	(0.728)	(0.710)	(0.703)	(0.705)	(0.703)	(0.888)	(0.762)	(0.877)	(0.709)
Vascular dementia	0.999	0.969	0.900	0.939	0.916	1.010	0.756	0.820	0.903
	(0.303)***	(0.314)***	(0.315)***	(0.301)***	(0.300)***	(0.339)***	(0.306)**	(0.310)***	(0.311)***
Other dementia	0.113	0.062	0.116	0.101	0.096	-0.086	-0.035	-0.005	-0.114
	(0.325)	(0.328)	(0.331)	(0.323)	(0.325)	(0.370)	(0.333)	(0.337)	(0.339)
Moderate perceived severity	0.425	0.440	0.363	0.411	0.398	0.113	0.392	0.321	0.249
	(0.466)	(0.470)	(0.474)	(0.467)	(0.471)	(0.517)	(0.471)	(0.487)	(0.474)
Severe perceived severity	0.474	0.438	0.283	0.403	0.364	-0.075	0.414	0.230	0.297
	(0.534)	(0.540)	(0.543)	(0.533)	(0.536)	(0.600)	(0.539)	(0.556)	(0.546)
Constant	-0.623	-0.549	-0.658	-0.582	-0.589	-1.487	-0.027	-0.843	-0.443
	(1.034)	(1.048)	(1.052)	(1.030)	(1.031)	(1.182)	(1.075)	(1.059)	(1.102)
Observations	282	274	268	287	285	227	269	259	266

Most of the coefficients on dummies are interpreted in relation to the reference category. The reference categories for carer characteristics are respectively: Male, Younger than 75 years old, Below university education, No financial difficulties, Main carer status, Duration of caring under 5 years. The reference categories for the care recipient characteristics are: Younger than 74 years old, Duration of symptoms under 11 years, Mild severity of dementia.

Robust standard errors in parentheses; ***=p-value<0.01, **=p-value<0.05, *=p-value<0.1

Table S12 – First-stage results on the instrument of the IV approach.

Instrumental Variable	ASCOT-Carer score	Self-efficacy on symptoms management	Self-efficacy on service use	Overall life satisfaction	Happiness yesterday	Overall costs	Carer's healthcare costs	Care recipient's healthcare costs	Care recipient's social care costs
Two-Stage Least Square estimator: first-stage estimated coefficient in main analysis									
Travel time	-1.314***	-1.242***	-1.303***	-1.267***	-1.284***	-1.376***	-1.354***	-1.444***	-1.277***
CD F statistic	48.153	41.741	41.138	45.057	45.341	47.155	49.597	56.661	43.216
Two-Stage Least Square estimator: first-stage estimated coefficient in sensitivity analysis									
Travel time	-1.119***	-1.088***	-1.145***	-1.127***	-1.123***				
County LA	0.418***	0.395***	0.428***	0.386***	0.399***				
London LA	0.052	-0.003	-0.015	-0.004	0.015				
Metropolitan LA	0.090	0.086	0.080	0.068	0.071				
CD F statistic	25.267	0.630	24.255	22.990	23.712				
SH test	0.187	0.889	0.112	0.783	0.441				

LA=local authority; CD F statistic=Cragg-Donald F statistic; SH test=p-value of the Sargan-Hansen test; *** p<0.01, ** p<0.05, * p<0.1.

Table S13 – IV results when the logit regression uses fewer covariates.

	Variable	ASCOT-Carer score	Self-efficacy on symptoms management	Self-efficacy on service use	Overall life satisfaction	Happiness yesterday	Overall costs	Carer's healthcare costs	Care recipient's healthcare costs	Social care costs
	Admiral nursing	1.206	1.692	3.048	0.126	1.022	78.250	-37.698	132.775	-339.518
		(0.832)	(2.980)	(2.519)	(0.644)	(0.641)	(430.475)	(182.731)	(226.054)	(317.961)
Carer characteristics	Female	-0.502	-2.440	-4.634	-0.247	-0.125	-28.544	202.795	1.039	-21.963
		(0.471)	(1.381)*	(1.223)***	(0.304)	(0.311)	(200.534)	(174.873)	(139.468)	(164.331)
	75 years old or older	0.330	-0.670	-3.701	0.657	0.540	-25.490	221.863	-14.220	35.980
		(0.504)	(1.541)	(1.216)***	(0.334)**	(0.342)	(233.544)	(188.263)	(169.471)	(169.726)
	Bachelor's degree	-0.536	-2.250	-2.561	-0.434	-0.462	395.374	-125.189	-107.790	376.839
		(0.451)	(1.353)*	(1.194)**	(0.282)	(0.299)	(205.187)*	(65.191)*	(92.935)	(148.976)**
	Master's degree and above	-0.651	-3.466	-2.169	-0.115	0.186	390.435	-11.330	-73.337	180.982
		(0.688)	(2.292)	(1.847)	(0.499)	(0.504)	(384.573)	(285.682)	(188.187)	(289.328)
	Retired	0.458	0.829	2.535	0.214	0.542	209.323	211.057	22.755	238.874
		(0.440)	(1.376)	(1.183)**	(0.284)	(0.304)*	(203.524)	(140.206)	(138.350)	(143.209)*
	Some financial difficulties	-1.609	-1.041	-3.332	-0.736	-1.006	23.628	-8.590	6.982	-1.702
		(0.536)***	(1.654)	(1.322)**	(0.341)**	(0.349)***	(200.511)	(84.422)	(118.033)	(147.276)
	Severe financial difficulties	-2.418	-2.033	-3.619	-1.284	-1.405	52.210	154.272	-103.427	330.809
		(0.562)***	(1.652)	(1.356)***	(0.352)***	(0.374)***	(218.317)	(152.217)	(123.662)	(186.860)*
	Prefer not to say financial difficulties	-2.903	-1.502	-4.510	-0.795	-1.218	731.766	393.986	398.984	240.235
		(0.753)***	(2.176)	(1.789)**	(0.427)*	(0.468)***	(433.377)*	(283.079)	(314.593)	(238.753)
	EQ-5D score	7.216	15.135	9.793	4.204	4.288	426.686	-417.874	-46.986	324.842
		(1.043)***	(3.521)***	(2.448)***	(0.886)***	(0.933)***	(392.938)	(430.666)	(271.094)	(344.914)
	Joint or not main carer	0.854	4.433	3.272	1.060	0.941	295.323	-142.682	173.247	110.006
		(0.868)	(1.748)**	(2.027)	(0.451)**	(0.467)**	(463.459)	(126.331)	(309.812)	(256.545)
	Total hours of care	-0.105	0.247	0.092	-0.022	-0.027	10.382	-11.737	12.825	-8.790
		(0.027)***	(0.082)***	(0.073)	(0.016)	(0.017)	(13.143)	(5.933)**	(8.752)	(9.244)
	Caring for 5 years or more	-0.468	0.150	0.112	-0.248	-0.083	-172.137	-165.672	-412.900	313.522
		(0.422)	(1.360)	(1.046)	(0.258)	(0.296)	(217.238)	(102.202)	(156.512)***	(161.671)*
	No replacement for a break	-1.348	-3.144	-2.525	-0.696	-0.751	24.609	-258.181	-115.666	85.345
		(0.421)***	(1.229)**	(1.031)**	(0.258)***	(0.268)***	(181.394)	(134.056)*	(127.202)	(136.017)
Care recipient characteristics	75 years old or older	0.405	2.800	2.688	0.081	0.094	160.927	43.467	203.690	-47.528
		(0.443)	(1.484)*	(1.177)**	(0.296)	(0.322)	(191.720)	(78.937)	(132.626)	(140.259)
	Symptoms for 11 years or more	0.869	3.929	0.424	0.506	0.733	321.304	-76.477	369.355	-129.195
		(0.626)	(2.125)*	(1.840)	(0.382)	(0.541)	(399.610)	(242.323)	(318.194)	(264.733)

Variable	ASCOT-Carer score	Self-efficacy on symptoms management	Self-efficacy on service use	Overall life satisfaction	Happiness yesterday	Overall costs	Carer's healthcare costs	Care recipient's healthcare costs	Social care costs
No diagnosis of dementia/don't know	-1.672	-7.381	-5.344	-0.099	-0.074	272.897	-178.472	202.151	-243.448
	(0.916)*	(2.229)***	(2.472)**	(0.432)	(0.493)	(608.973)	(223.001)	(538.307)	(310.076)
Vascular dementia	-0.927	-1.187	-1.561	-0.075	0.021	-6.534	24.100	211.136	-107.603
	(0.455)**	(1.405)	(1.181)	(0.296)	(0.310)	(199.681)	(118.761)	(118.339)*	(147.352)
Other dementia	-0.636	-0.410	-3.625	-0.059	0.179	20.426	158.149	246.435	-112.455
	(0.432)	(1.467)	(1.145)***	(0.313)	(0.329)	(224.680)	(171.690)	(174.690)	(171.303)
Moderate perceived severity	-0.660	-2.493	-3.664	-0.559	-0.868	-182.157	47.465	-163.398	44.009
	(0.613)	(1.801)	(1.509)**	(0.392)	(0.415)**	(284.872)	(105.708)	(189.087)	(172.782)
Severe perceived severity	-2.037	-4.358	-4.017	-1.261	-1.543	546.178	477.390	94.181	561.977
	(0.689)***	(2.158)**	(1.810)**	(0.472)***	(0.497)***	(357.364)	(253.730)*	(245.877)	(219.377)**
Constant	8.933	17.963	22.821	3.335	3.257	65.113	332.547	118.415	43.584
	(1.428)***	(4.407)***	(3.333)***	(1.099)***	(1.170)***	(558.894)	(539.411)	(408.036)	(424.088)
Observations	281	273	267	286	284	227	268	258	266
R-squared	0.415	0.224	0.287	0.318	0.285	0.134	0.145	0.112	0.100

Most of the coefficients on dummies are interpreted in relation to the reference category. The reference categories for carer characteristics are respectively: Male, Younger than 75 years old, Below university education, No financial difficulties, Main carer status, Duration of caring under 5 years. The reference categories for the care recipient characteristics are: Younger than 74 years old, Duration of symptoms under 11 years, Mild severity of dementia.

Robust standard errors in parentheses; ***=p-value<0.01, **=p-value<0.05, *=p-value<0.1

Figure S1 – Distribution of HRQoL and total hours of informal care.

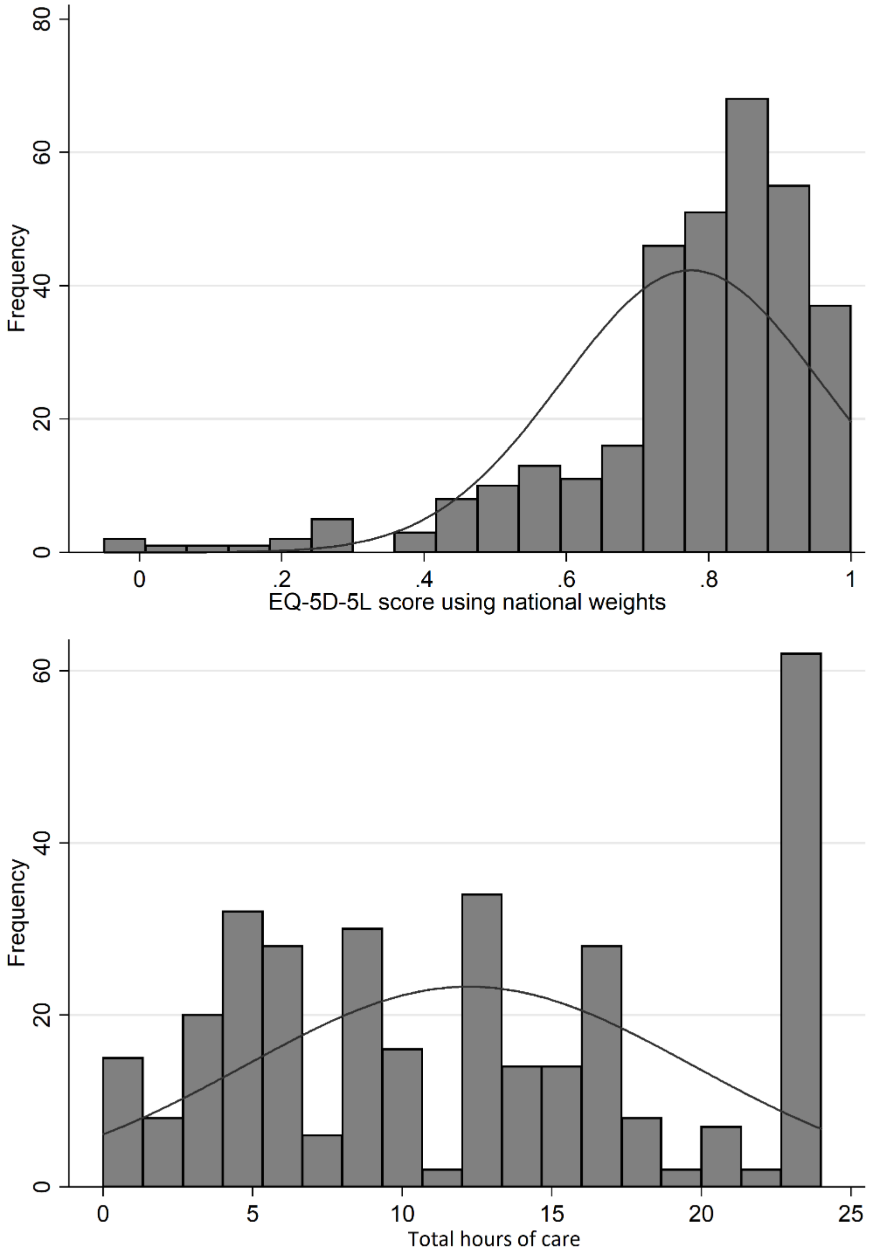

Figure S2 – Standardised difference in covariates before and after matching.

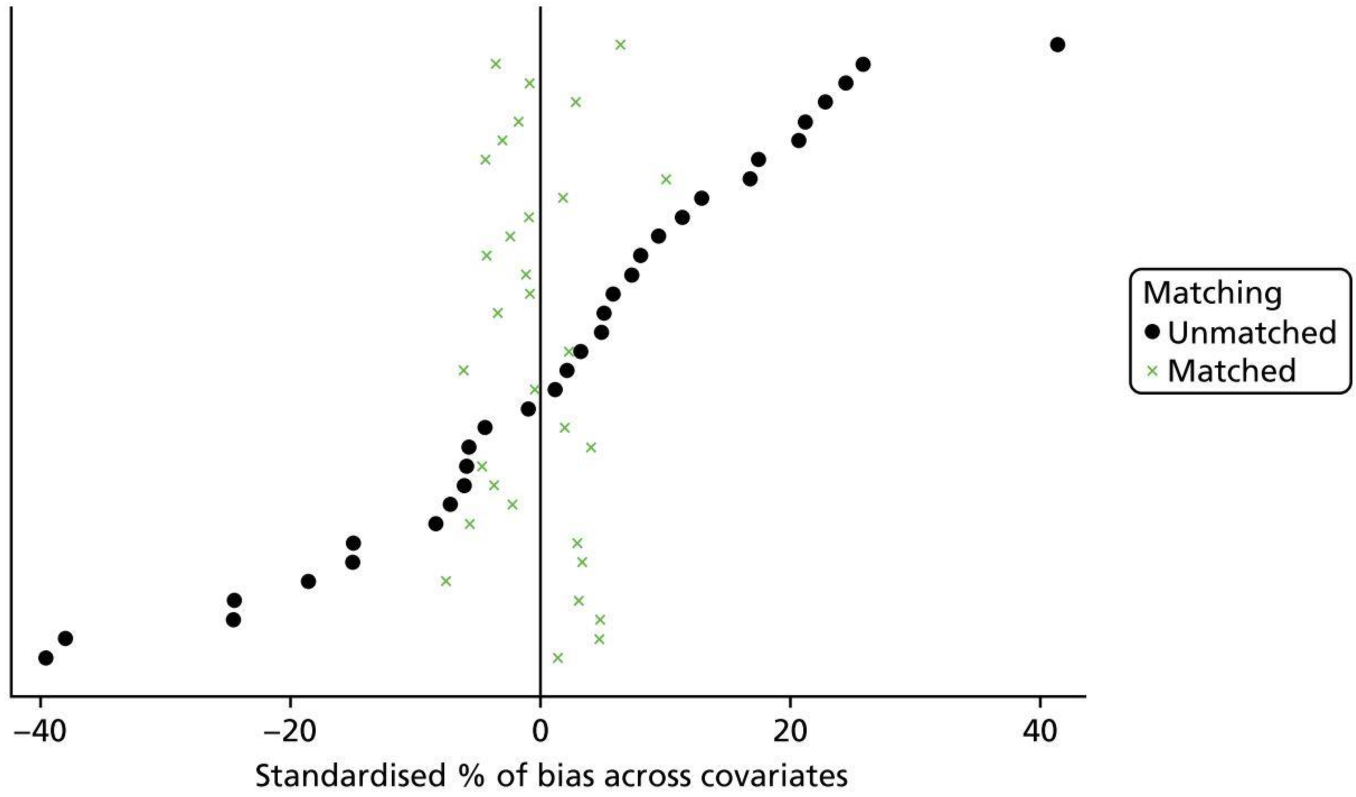

Figure S3 – Propensity score overlap before and after matching.

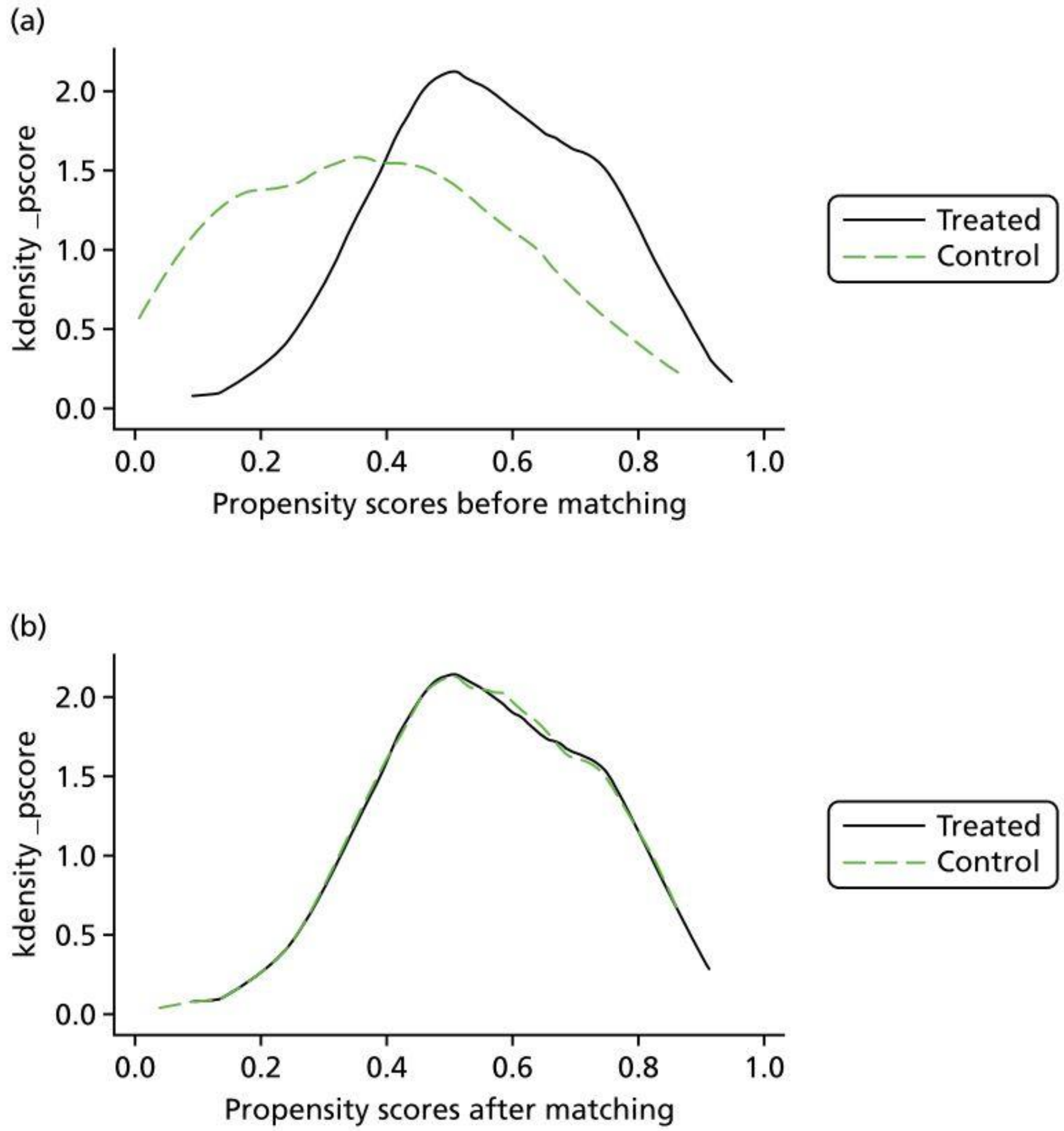

Figure S4 – Distribution of travel time.

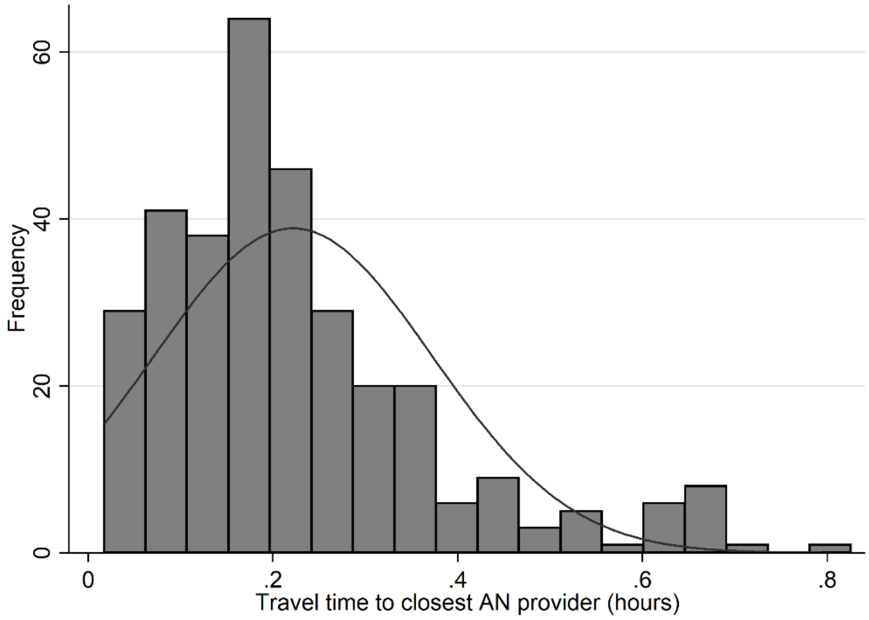

Supplement: Supplementary file 2 [file HSC-27-e734-s002.pdf]
